# The Origin of Large-Bodied Shrimp that Dominate Modern Global Aquaculture

**DOI:** 10.1371/journal.pone.0158840

**Published:** 2016-07-14

**Authors:** Javier Robalino, Blake Wilkins, Heather D. Bracken-Grissom, Tin-Yam Chan, Maureen A. O’Leary

**Affiliations:** 1 Department of Anatomical Sciences, HSC T-8 (040), Stony Brook University, Stony Brook, New York, United States of America; 2 Department of Biology, Florida International University, Biscayne Bay Campus, North Miami, Florida, United States of America; 3 Institute of Marine Biology and Center of Excellence for the Oceans, National Taiwan Ocean University, Keelung, Taiwan, Republic of China; Institute of Botany, CHINA

## Abstract

Several shrimp species from the clade Penaeidae are farmed industrially for human consumption, and this farming has turned shrimp into the largest seafood commodity in the world. The species that are in demand for farming are an anomaly within their clade because they grow to much larger sizes than other members of Penaeidae. Here we trace the evolutionary history of the anomalous farmed shrimp using combined data phylogenetic analysis of living and fossil species. We show that exquisitely preserved fossils of †*Antrimpos speciosus* from the Late Jurassic Solnhofen limestone belong to the same clade as the species that dominate modern farming, dating the origin of this clade to at least 145 mya. This finding contradicts a much younger Late Cretaceous age (ca. 95 mya) previously estimated for this clade using molecular clocks. The species in the farmed shrimp clade defy a widespread tendency, by reaching relatively large body sizes despite their warm water lifestyles. Small body sizes have been shown to be physiologically favored in warm aquatic environments because satisfying oxygen demands is difficult for large organisms breathing in warm water. Our analysis shows that large-bodied, farmed shrimp have more gills than their smaller-bodied shallow-water relatives, suggesting that extra gills may have been key to the clade’s ability to meet oxygen demands at a large size. Our combined data phylogenetic tree also suggests that, during penaeid evolution, the adoption of mangrove forests as habitats for young shrimp occurred multiple times independently.

## Introduction

Very few of the more than 3,000 known marine decapod shrimp species [1] have been the targets of industrial-scale aquaculture. To be a high priority for farming, a shrimp species must have two features—large body size and a behavioral preference for warm shallow waters. Tropical shrimp species that grow to very large sizes defy ecological and physiological patterns such as the temperature-size rule, which predict that warm environments select for small body sizes [2,3]. How the large-bodied tropical shrimp used in farming evolved has remained unknown, because the phylogenetic history of penaeoidean shrimp has never been examined in the context of molecular, morphological and fossil evidence.

Human exploitation of large shrimp species through industrial aquaculture in tropical areas has expanded extremely rapidly in the last five decades, and has been heavily criticized for the destruction of vulnerable coastal habitats, particularly mangrove forests [4,5]. Paradoxically, these same mangrove forests are hypothesized to be key nursery environments for natural shrimp populations [6,7]. In light of ongoing environmental degradation and global warming, it is particularly important to elucidate the ecological context in which valuable species such as these shrimp evolved.

## Results and Discussion

### Large body size in the ancestor of farmed shrimp

**Fig 1:**
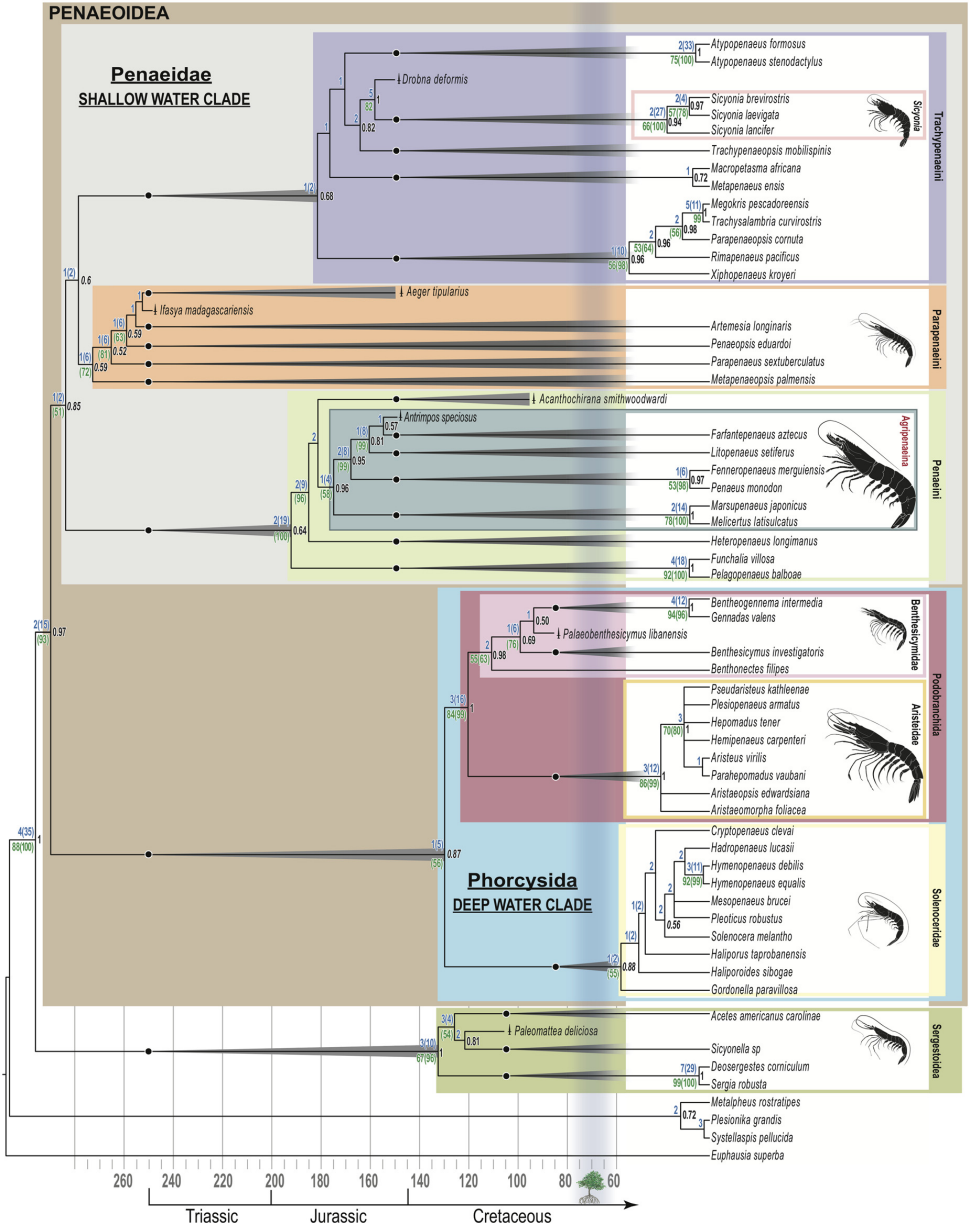
Minimum age phylogenetic tree of Penaeoidea. Agripenaeina, the clade of farmed shrimp, acquired a large body size despite the physiological constraints of their warm and shallow-water habitats. This clade is at least 145 my old because it includes a Late Jurassic species (^**†**^*Antrimpos speciosus*) that inhabited the warm waters preserved in the Late Jurassic Solnhofen limestone [14,15]. Ecological associations between shallow-water penaeoideans and mangrove forests occurred independently more than once, as clades of mangrove-associated shrimp (e.g., Agripenaeina and Trachypenaeini) predate the proposed Late Cretaceous origin of modern mangroves [16]. Shrimp silhouettes illustrate differences in maximum body size for each clade. Topology shown emerges from parsimony (strict consensus of 24 trees) and Bayesian analyses, with some Bayesian incongruences noted in the Extended Results. Topology mapped to stratigraphic record with range extensions (cones) dictated by fossil placements (black dots indicate first appearance datum in the stratigraphic record) [17]. Bremer Support (blue) and jackknife values over 50% (green) are indicated. Values in parentheses were calculated without fossil taxa (fossil exclusion produces a congruent tree). Bayesian posterior probabilities in black, with values italicized for clades that are congruent except for the placement of ^†^*Aeger tipularius*, which occupies a different position in the Bayesian tree (see S4 Fig). Tree icon and vertical shading indicate earliest evidence of modern mangroves [16].

We assembled a combined-data phylogeny of living and fossil Penaeoidea (to which the Penaeidae belong) to examine the pattern and timing of the evolution of industrially farmed shrimp and to investigate the origin of large body size and its relationship to key anatomical features and behaviors. Our new large-scale dataset of anatomical and behavioral characters (assembled in Morphobank [8]), was combined with newly generated and previously published molecular sequences [9] to conduct the first analysis of this clade that integrates phenomic data and fossil taxa into the phylogeny. Thirty-seven percent of the characters in this combined dataset are new compared to previously published work [9]. To facilitate discussion of the evolutionary implications of our combined data tree (Fig 1), we provide new names for clades that have not been named in previous work (phylogenetic definitions of key clades are also shown in S1 Table, and the etymology of all new names is discussed in the Materials and Methods section).

Penaeoidea split basally into two clades (Fig 1): clade Phorcysida (new name) with a marked preference for deep water, and clade Penaeidae with preference for shallow waters (bathymetric behavior optimization shown in S1 Fig). This split has been proposed before by taxonomists [10] and by recent molecular phylogenies [9]. Within the shallow water clade (Penaeidae), the sub-clade Agripenaeina (new name) contains all species that are farmed at an industrial scale, including the two species that currently dominate global farming, *Litopenaeus vannamei* and *Penaeus monodon* [11]. Shallow water behaviors allow these shrimp to be grown in low-cost coastal impoundments with relatively small water volumes [12]. By optimizing body size onto the penaeoidean tree (S2 Fig) we found evidence for the derived acquisition of large body size in the ancestor of the clade Agripenaeina. Most other members of Penaeidae also live in shallow tropical waters, but they are not farmed at an industrial scale because they have smaller bodies and are less valuable in the global shrimp market ([13], clades Parapenaeini and Trachypenaeini in Fig 1). Other instances of large body size are found elsewhere across the broad clade Penaeoidea (in clade Phorcysida, S2 Fig), but these species are not suitable for tropical coastal farming because they live in cold and deep waters (S1 and S3 Figs).

**Fig 2:**
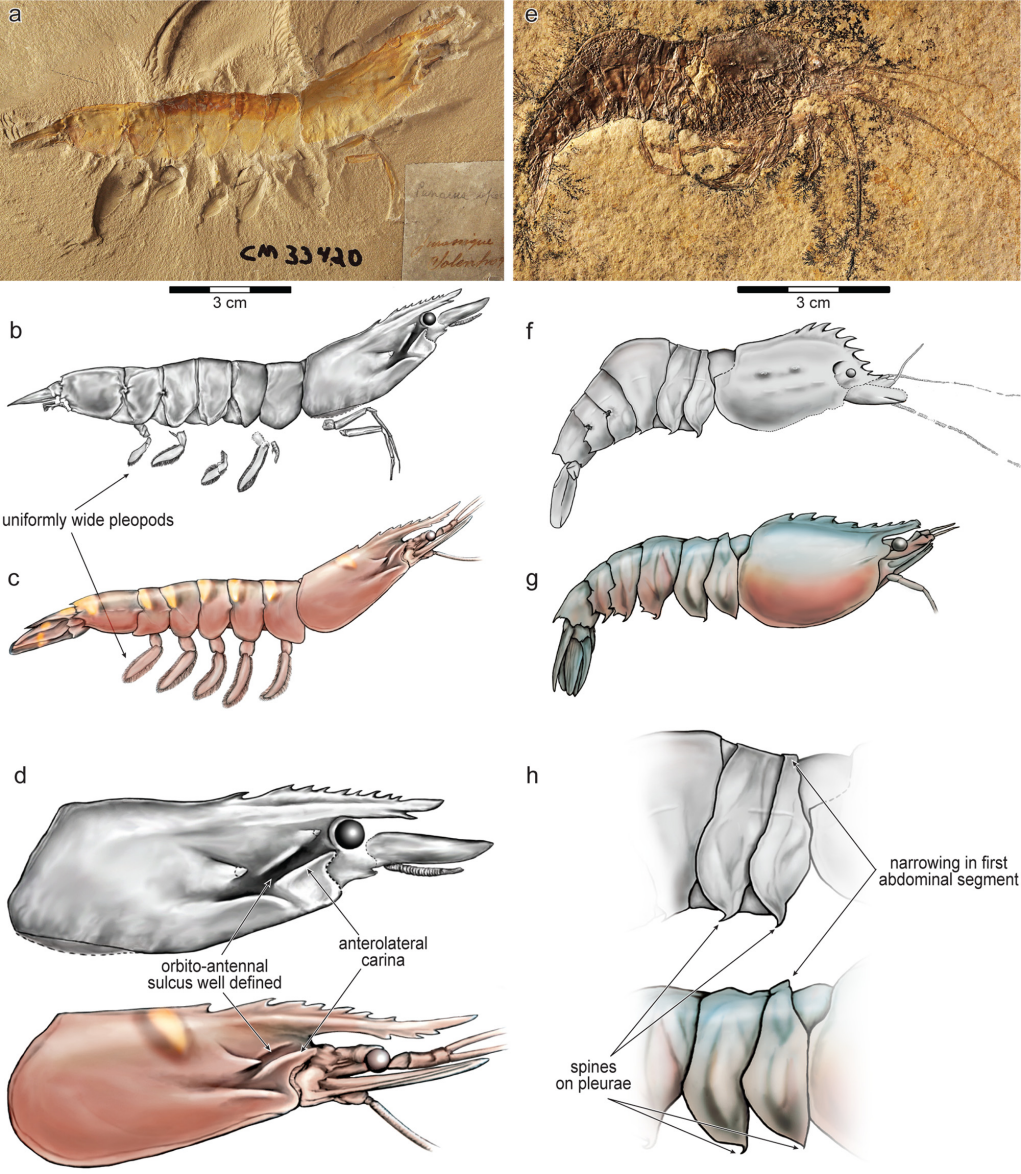
Members of Agripenaeina and Trachypenaeini from the Late Jurassic. ^†^*Antrimpos speciosus* (panels **a** and **b**, CM-33420) and ^**†**^*Drobna deformis* (panels **e** and **f**, CM-29467), fossils from the Solnhofen limestone, Germany (ca. 145 mya [18]) preserve key features that link them phylogenetically to shallow-water penaeoideans. ^†^*Antrimpos speciosus* belongs to Agripenaeina, the clade accounting for 90% of shrimp farmed for human consumption, and is shown in comparison to *Penaeus monodon* (**c**, giant tiger shrimp), a living member of Agripenaeina. ^**†**^*Drobna deformis* is the sister taxon of *Sicyonia* (Trachypenaini), and is shown in comparison to the living *Sicyonia lancifer* (**g**, rock shrimp). **d** and **h** show derived features shared by fossil and living shrimp. Additional synapomorphies for all clades are listed in S2 and S3 Tables. Scale bars = 3 cm.

Along with body size, derived anatomy such as distinctive carapace ornamentation and swimming legs that are wide along their entire length, characterize the clade Agripenaeina (S2 and S3 Tables). Exquisitely preserved specimens of the fossil †*Antrimpos speciosus* (Fig 2) from the Solnhofen limestone of Germany (Late Jurassic [14,18]) show derived characters that support placement of this extinct species inside the clade Agripenaeina, by both parsimony and Bayesian methods (Figs 1 and S4). This relationship means that the farmed clade, Agripenaeina, of large-bodied, tropical, shallow-water shrimp dates at least to the Late Jurassic, approximately 145 mya [18] (Fig 1). This result is in contrast to a Late Cretaceous age (ca. 95 mya) previously estimated for Agripenaeina using molecular clocks [9]. The occurrence of a member of Agripenaeina in the Solnhofen limestone suggests that shallow, warm-water preferences were already established in this clade by the Late Jurassic, because the paleoenvironment that created the Solnhofen limestone is interpreted to have been a sub-tropical, shallow-water coastal area, protected from the open ocean by sponge mounds and coral reefs [15]. Marine fossils found there are hypothesized to have been carried from the surrounding shallow waters into an isolated lagoon, where hypersaline and hypoxic conditions favored preservation [15]. General similarities between †*Antrimpos speciosus* and living members of Agripenaeina were noted by paleontologists almost a century ago [19,20], but we are the first to test this relationship phylogenetically.

The position of †*Drobna deformis*, a second fossil species from the Solnhofen limestone [14] shows that, like the large-bodied clade Agripenaeina, the smaller-bodied clade Trachypenaeini also had members that inhabited the warm waters of the Tethys Sea during the Late Jurassic [15]. †*Drobna deformis* is the sister taxon of the rock shrimp clade *Sicyonia* (Trachypenaeini, Fig 1). Derived characters such as a short carapace and distinctive ornamentation on the abdomen support this relationship (Fig 2 and S2 Table). Thus, Agripenaeina and Trachypenaeini have each been committed to warm and shallow waters since they separated more than 250 mya (Fig 1), as determined by our phylogenetic optimization of bathymetric and thermal behavioral preferences across Penaeoidea (S1 and S3 Figs). Despite their similar behaviors, these two clades are conspicuously different in their body sizes and in their value to aquaculture.

### Challenges of becoming large in warm waters

Given the ecological similarities (shallow waters and warm temperatures) under which the clades Agripenaeina and Trachypenaeini now live and appear to have lived since at least the Late Jurassic, it is especially relevant to ask what important anatomical differences might account for the very different growth potentials of these clades. Body size is not only an important trait in aquaculture, it is also a key biological property influencing organismal function, ecology, and evolution [21]. Patterns observed in nature suggest that warm waters, such as those inhabited by modern shallow-water shrimp, select for small body sizes. Penaeids and many other aquatic species have smaller adult sizes in low latitudes with warm waters, while large adult sizes are more common in high latitudes with cool waters [22–24]. Reductions in body size have also been found to track the rise of ocean temperatures caused by global warming [25,26], leading to the suggestion that size reductions might become a widespread global consequence of future climate change [26]. On an evolutionary timescale, increases in body size with cooling water temperatures have also been documented in the fossil record of deep-water ostracods (small crustaceans known as seed shrimp [27]).

Laboratory observations of the phenomenon known as the temperature-size rule (TSR) also corroborate the hypothesis that smaller body sizes are favored in warm aquatic habitats. The TSR refers to the tendency of ectothermic organisms to reach maturity faster and at smaller body sizes when reared at higher temperatures [2], and is considered one of the most widespread phenomena in nature [2,28]. A mismatch between the accelerated metabolism that comes with higher temperatures and the finite oxygen supplies available for these metabolic demands has been proposed to explain the TSR [29]. When temperatures are high, growth is fast and oxygen consumption is high. Final adult sizes are small, however, because at a critical point most organisms are unable to obtain sufficient oxygen to support both baseline metabolism and additional growth [30,31]. The TSR is more marked in water-breathing than in air-breathing organisms [28], presumably because adequate amounts of oxygen are much harder to obtain in warm water than in warm air (air carries much more oxygen than water, and diffusion rates for oxygen are much higher in air than in water [31]). Hypoxia alone has been shown to be sufficient to cause smaller body sizes in a variety of ectothermic species (reviewed in [32,33]), including *Litopenaeus vannamei* and *Penaeus monodon*, two farmed species from the clade Agripenaeina [34].

Thus patterns in nature and in the laboratory indicate that living in warm water habitats should result in selection of smaller-bodied species, and that one plausible contributing mechanism for this effect is the unmatched oxygen demands that occur at high temperatures. Oxygen has been recognized for a long time to be an important factor in the evolution of animal body size [35,36]. Analyses of the fossil record have shown that increases in body size in organisms ranging from bacteria to animals and plants are well-correlated with increases in atmospheric oxygen throughout Earth’s history [37–41]. Given these physiological and evolutionary trends, we used our combined data phylogeny to look for respiratory transformations that may have been related to the anomalous acquisition of large body size in the warm water Agripenaeina shrimp.

### A larger breathing apparatus preceded large body size in the farmed clade

As described above, our phylogeny, interpreted in light of physiological principles and of broad evolutionary patterns, predicts respiratory differences between the Agripenaeina and smaller-bodied penaeids. Optimization of branchial anatomy across Penaeoidea indicates that the Agripenaeina have more gills and epipods (the branchial structures that participate in gill cleaning [42]) than do most other shallow-water species. Extra gills and epipods appear to have been retained evolutionarily in Agripenaeina and their close relatives (clade Penaeini), while other shallow-water clades reduced the numbers of these structures when they diverged from the farmed clade more than 250 mya (Fig 1 and Table 1). Among the closest relatives of the large-bodied Agripenaeina are species of *Funchalia* and *Pelagopenaeus* (Fig 1), which have the same number of gills and epipods as the farmed species, yet their body size is smaller (S2 Fig). *Funchalia* and *Pelagopenaeus* are, however, also behaviorally very different from the Agripenaeina because they are pelagic, and undergo long vertical migrations to reach very deep waters (below 1,000 m, with reports of occurrences below 5,000 m, S1 Fig) [43–45]. This observation is consistent with the finding that the largest number of gills and epipods in all of Penaeoidea is found in deep-water species (see below). Our data indicate that the closest living relative of Agripenaeina, *Heteropenaeus longimanus*, is the only known shallow-water, small-bodied penaeid species that has as many gills and epipods as the farmed shrimp.

**Table 1:** Gills and epipods in Penaeoidea. Among the shallow-water clades, Agripenaeina (and other members of Penaeini) have the highest number of gills and epipods. Deep-water clades have the highest numbers across all of Penaeoidea. Listed are the total numbers of gills and epipods per side, based on the ancestral states for each clade, using parsimony. App = associated appendix, Max = maxilliped, Per = pereiopod.

		Phorcysida (deep-water)		Penaeidae (shallow-water)		
Segment	App	Podobranchida	Solenoceridae	Penaeini (inc. Agripenaeina)	Parapenaeini	Trachypenaeini
VII	Max 1	1	1	1	1	1
VIII	Max 2	3	3	3	3	3
IX	Max 3	4	3	3	3	3
X	Per 1	4	3	3	3	3
XI	Per 2	4	3	3	3	3
XII	Per 3	4	3	3	3	3
XIII	Per 4	3	3	2	2	1
XIV	Per 5	1	1	1	0	0
TOTAL GILLS		24	20	19	18	17
EPIPODS		7	7	6	5	5

Thus with the key exception of the large-bodied Agripenaeina, large numbers of gills and epipods are primarily a characteristic of penaeoids with preferences for deep waters. Deep-water behaviors in Penaeoidea involve swimming across a long vertical range [46–49], which requires traveling through waters that are very cold and viscous, with reduced oxygen bioavailability because of the low diffusion rates in cold pressurized water [30,31]. Thus although deep waters are often rich in oxygen, deep-water penaeoids may face significant challenges in extracting this oxygen. The largest numbers of gills and epipods are found in Podobranchida, which includes species that reach abyssal zones (deeper than 4,000 m, S1 Fig). Second to Podobranchida in gill number are the Solenoceridae, which is a deep-water group that reaches into bathyal zones (deeper than 1,000 m, S1 Fig). The Agripenaeina are fundamentally different from these deep-water clades, because they have a range that is restricted to very shallow and warm waters (S1 and S3 Figs).

### Mangrove forests, the shrimp fossil record and global warming

As previously noted, a molecular clock analysis of Penaeoidea estimated a Late Cretaceous origin (ca. 95 mya) for the clade Agripenaeina [9]. Although not discussed in that paper, such an estimate would have made the origin of the Agripenaeina approximately contemporaneous with the hypothesized origin of modern mangrove forests based on current fossil evidence [16], a significant finding because living members of Agripenaeina have close ecological associations with estuarine mangroves [6,50–52]. Inclusion of exquisitely preserved Late Jurassic shrimp fossils in our phylogenetic analysis, however, indicates that the minimum age of the clade Agripenaeina is at least 145 my (Fig 1), a date that is much older than the currently recognized Late Cretaceous origin for mangroves [16]. This result implies that adopting mangroves as a habitat occurred multiple times independently within the clade Agripenaeina. Members of the small-bodied, shallow-water clade, Trachypenaeini, also use mangroves as part of their life cycle (e.g., species of *Metapenaeus* [6]). Our tree suggests that the Trachypenaeini independently evolved a relationship with mangroves relative to that of the Agripenaeina because these two lineages diverged from each other at least 250 mya (Fig 1), far earlier than modern mangroves are thought to have appeared [16]. The convergent acquisition of mangrove habitat use by multiple lineages of tropical shrimp underscores the ecological importance of these forests, and the urgent need to protect them, as they are disappearing at rapid rates globally [5].

Global seafood production is on track to be one critical means by which humans will meet the demand for protein of an expanding 21^st^ century population [53]. Climate change is already expected to impact seafood production by altering species distributions, reproductive patterns, and trophic relationships [54,55]. A new and serious concern derived from modeling studies and ecological surveys is that ocean warming may cause widespread reductions in the size of marine species [25,26,56]. Warming is also predicted to cause reductions in total oceanic oxygen and the expansion of oxygen-depleted zones [57,58]. Thus the current central role of Agripenaeina in global seafood production may be at risk, considering that their uniquely large body size (unique among shallow-water penaeoids) may be closely linked to their respiratory capacity. Less overall oxygen in much of the ocean, coupled with higher metabolic demands caused by higher temperatures could potentially constrain growth and affect survival of these farmed shrimp. Tropical shrimp populations may also shift towards higher latitudes, as has been recorded for many fish populations already [59]. The reliance of aquaculture on such a taxonomically restricted group of exceptional species (only two species of Agripenaeina contribute more than 90% of all shrimp farmed in the world, S4 Table) makes this key food resource especially vulnerable to these threats.

## Materials and Methods

### Specimens

No collection permits were required for this study, as all specimens studied are vouchered museum specimens. Specimen information is further detailed in this Materials and Methods section and in S5 Table.

### Taxonomic scope

A total of 63 species-level terminal taxa were sampled (S5 Table): 48 species representing 44 different genera in Penaeoidea (out of the 57 genera currently assigned to the group [1,60,61]). Four extant members of Sergestoidea, three members of Caridea, and one member of Euphausiacea were sampled as outgroups. Six fossil species taxonomically attributed to Penaeoidea and one to Sergestoidea [60] were also sampled. Our sample spans the five major taxonomic groups in the living Penaeoidea [61], and includes eight out of nine genera in Aristeidae, four out of five genera in Benthesicymidae, nine out of 10 genera in Solenoceridae, 22 out of 32 genera in Penaeidae, and the single genus in Sicyoniidae.

More than 100 extinct species have been taxonomically assigned to the superfamily Penaeoidea [60]. However, many of these species attributions are supported only by sparse evidence, sometimes from only one or a few poorly preserved specimens (e.g. [62,63]). We only included in this study fossils that were 1) accessible for direct observation, 2) represented by well-preserved, relatively complete specimens, 3) broadly representative of penaeoidean morphological diversity, and 4) representative of the stratigraphic range that Penaeoidea is hypothesized to span. The only exception was ^†^*Paleobenthesicymus libanensis*, for which only one partial specimen was available to us for study (S5 Table). However, ^†^*P*. *libanensis* has been recently re-described thoroughly [64], including the direct interpretation of important characters, with extensive photographic documentation. This published description allowed sufficient character documentation for inclusion in our analysis. Because the taxonomy of many penaeoid fossils in collections around the world is uncertain (see [65] for example), we used only fossils from known localities, and scored characters from the smallest possible number of specimens that preserved diagnostic features of the species (to avoid chimeric taxa). Fossils sampled are in collections of the National Museum of Natural History (USNM), Carnegie Museum of Natural History (CM), American Museum of Natural History (AMNH), and Museo Civico di Storia Naturale di Milano (MSNM). Details on scored specimens, missing data, and geological ages are summarized in S5 and S6 Tables.

### Phenomic characters

Phenomic characters were defined, coded, and organized using the web application and database MorphoBank (http://www.morphobank.org) [8]. A total of 339 characters representing adult anatomy and behaviors were collected primarily from direct observation of vouchered museum specimens (extant and fossil), supplemented with records from the literature. Complete specimen information for each taxon scored is listed in S5 Table. The phenomic matrix, including character descriptions and photographs documenting homology is publicly available in MorphoBank under Project 2282.

We culled characters from published taxonomic keys and prior phylogenetic studies [10,61,66,67] to assemble a list of adult anatomical and behavioral characters for penaeoidean phylogeny. We edited published characters to eliminate redundancy and to apply reductive coding [68] to character definitions. Reductive coding permits maximal homology grouping for the state ‘present’ when a character has multiple variations of the ‘present’ state. New characters were also defined by us and added to the matrix in the course of observing the anatomical diversity of specimens. The 339 phenomic characters were treated as discrete and unordered.

For characters describing the relative size of structures, a threshold reflective of observed extremes of variation was used to guide scoring. All characters were scored from fully developed adult specimens. Whenever polymorphisms were observed or documented in the literature (e.g., rostrum length [character 1] in some Aristeidae), character states were scored as polymorphic. Adult anatomical characters describe features of the eyes, antennules, mouthparts, gill formulae, pereiopods (thoracic legs), carapace ornamentation, abdominal cuticle, pleopods (abdominal legs), uropods, and telson. Hundreds of new images (accessible at MorphoBank, Project 2282) were used to document museum specimens used for scoring, and to justify character states assigned to extant and fossil taxa.

Some characters were modified relative to their original descriptions from the literature, as observations of specimens revealed the existence of variation that had not been explicitly documented previously. We found that morphological characters have rarely been documented comparatively across Penaeoidea (with some notable exceptions in [61,66]). This has resulted in ambiguity regarding terminology and definitions of morphological features, and thus revisions to the definition of some characters were necessary. In most cases we maintained the original terminology used by previous authors, and, in cases of ambiguity between different authors, we typically followed the terminology of Perez-Farfante and Kensley [61]. We also found little consistency across the paleontological literature regarding anatomical terminology, and very little overlap between the paleontological and neontological literature in this respect. This lack of consistency hindered the use of published descriptions of some fossil species for scoring. Thus, as noted above we scored fossil characters directly from original specimens.

Carapace ornamentation, particularly ridges and depressions (carinae and sulci respectively), have been used extensively in penaeoidean taxonomy (e.g., [61]). Coding of these characters was particularly challenging due to the degree of variation, the vague definitions in the literature in some cases, and the complexity of these features. Across the taxonomic scope of our study, carinae and sulci that appear to fit different definitions can occupy approximately similar locations on the shrimp carapace. To score these features objectively and consistently across taxa, we used structures that are widely conserved (often invariant) as spatial landmarks, and established explicit boundaries for each feature (details can be found in MorphoBank, Project 2282). We also used character ontologies as defined in MorphoBank [8]. These are explicit rules defined by us that specify the interrelatedness of characters, for example: if one character is scored absent, the subsequent (dependent) character must be scored inapplicable. Using these ontologies greatly aids in detecting potential scoring errors. Some of the ontological relationships defined in our matrix represent deviations from previously proposed character definitions. In such cases our notes in MorphoBank provide explanations to justify these relationships (Project 2282).

Most of the scores for extant terminal taxa were determined by direct observations of vouchered specimens from the Department of Invertebrate Zoology, National Museum of Natural History (USNM) collections (S5 Table) and scored cells in the matrix can be traced to these specimens. When poor preservation of specimens did not allow for direct scoring, we obtained character state information from the literature and this is also noted in the matrix cells in MorphoBank. Missing data for features that exist but are not preserved in museum specimens and not available from the literature were scored using the MorphoBank convention ‘not presently available’ (NPA) and are treated as ‘?’ in the final analysis. Missing data for features that are inapplicable, for example, the size of an exopod in taxa lacking an exopod, are treated as ‘?’ in the final matrix. Characters were scored at the species level, without assumptions of genus-level monophyly. Sperm ultrastructure characters were scored exclusively from the literature [69–75] and are the only character set (16 characters) for which we assumed synapomorphy at the genus-level, because sperm morphology has been recorded for only very few species. One character associated with female genitalia (thelycum type, character 299) is scored following genus-wide statements published in [61], which are understood to be applicable to all members of each genus. The percentages of missing and inapplicable data for each taxon in our matrix are shown in S5 Table.

### Notes on the coding of three key phenomic characters

An important objective of our study was to optimize the character body size onto the phylogeny of Penaeoidea, because the large size of farmed shrimp is important for their suitability for farming, with small-bodied species generally being considered of low commercial value [76]. Body size has also been recognized as an extremely important variable in evolutionary biology (e.g., [21]). Unfortunately, the most detailed studies on growth dynamics are restricted to species of the farmed clade, Agripenaeina (e.g. [77]), and the true growth potential of most penaeoids has not been firmly established. Surveys that assess body size in large natural samples are usually restricted to populations considered a priori of potential significance to fisheries. For these reasons, direct quantitative comparison of mean body size as a continuous character (including a range of variation), was not feasible given currently available data. That said, as described below, there are a number of sources recording the maximum body size of many penaeoidean species as seen in the wild.

Our measure of body size was maximum body length (character 316), because these data were available from two detailed assessments of penaeoid species that focused on their value (or lack thereof) to fisheries [76,78], and because this variable could be scored in extinct species. We supplemented these sources with other data collected from the literature. Specific references supporting cell scores can be found in MorphoBank, Project 2282. Because body size data might be considerably incomplete for some species, we did not attempt to make subtle distinctions regarding this character. Instead, we defined only three character states: small species measuring up to 5 cm; medium sized species with a very broad range exceeding 5 cm but not reaching 20 cm in total length (which accommodates most penaeoids); and large species, cases in which the shrimp are known to reach or surpass 20 cm in total length. Such broad categories provide a conservative assessment of the occurrence of very large sizes among shrimp. Body sizes used in all cases are those observed in natural populations, regardless of sex or frequency of occurrence. For fossil species, we used data reported in the literature from vouchered museum specimens, and, when available, supplemented these data with approximations from direct observation of fossils. In Fig 1, the body sizes illustrated correspond to the median of the three character states defined above (2.5 cm, 12.5 cm, and 27.5). The median of the large category (27.5 cm) was set from a maximum body length reported for a penaeoid of 35 cm (*Penaeus monodon* [76,78]).

A second noteworthy character used in our matrix is maximal depth occupied by individual species. Penaeoids have significant swimming ability, which they use to migrate vertically and horizontally in the water column. Variables that are known to influence these species-specific movements include substrate, salinity, temperature, turbidity, and oxygen [52,79,80]. The depth of water that penaeoids occupy is therefore a taxon-specific behavior influenced by habitat preferences, and there are marked differences among species and groups of species (raw bathymetry habitat data for the taxa sampled in this study are listed in MorphoBank, Project 2282). For depth limits, we adopted boundaries that have been generally proposed as biologically relevant, based on oceanographic parameters and zonation of ocean fauna [81–84]: sub-littoral zone (continental shelf, 0 to -200 m), mesopelagic zone (-201 to -1,000 m), bathyal zone (-1,001 to -4,000 m), and abyssal zone (less than -4,000 m). We note that the term mesopelagic as a character state is used only in reference to the water depth at which some penaeoids are found, and is not meant to imply an exclusively pelagic behavior of a species. Bathymetry data were obtained from the USNM specimen collection database, The Encyclopedia of Life (EOL, http://www.eol.org), which integrates data from research efforts such as the Ocean Biogeographic Information System and from published scientific literature, and from bibliographical compilations [76,78]. References to data sources can be found linked to specific scores in MorphoBank, Project 2282.

Finally, penaeoid shrimp also display species-specific temperature preferences that affect their distribution and behavior [52,79,85,86]. Members of Agripenaeina for example have been described as stenothermic, with narrow temperature ranges of 15–20°C [79]. The critical thermal minima for some species in this clade have been experimentally determined to be around 6–8°C depending on acclimation temperature [87,88]. Therefore, we used 7°C and 15°C as boundaries to define three states for the character “minimum water temperature” (character 322): very cold waters below 7°C, cold to temperate waters between 7°C and 15°C, and waters warmer than 15°C.

Temperature data were obtained from the USNM specimen collection database, The Encyclopedia of Life (EOL, http://www.eol.org), which, as noted above integrates data from research efforts and published scientific literature, and from bibliographical compilations [76,78]. References to data sources can be found linked to specific scores in MorphoBank, Project 2282.

### Molecular characters

Histone H3 (H3), Sodium/Potasium ATPase alpha subunit (NAK), and Phosphoenol-pyruvate kinase (PEPCK) formed the molecular partition for the phylogenetic analysis (1,480 characters). Previously reported sequences were obtained directly from the National Center for Biotechnology Information (NCBI) as detailed in S5 Table. New sequences were obtained from fresh or archival specimens with the following protocol: Genomic DNA was extracted from abdominal muscle, pleopods, or pereiopods using QIAGEN DNeasy Blood and Tissue Kit according to the manufacturer’s instructions. PCR amplification reactions were performed in 26 μL containing 2μL of DNA template, 6.45 μL of sterile water, 5 μL of 5x combinatorial PCR enhancer solution (CES) [89], 3 μL of 2 mM deoxyribonucleotide triphosphate mix (dNTPs), 2.5 μL of 10 x PCR Buffer, 2.3 μL of 5 M betaine, 2 μL of each 10 μM forward and reverse primer, 1.5 μL of 0.1 g/ mL bovine serum albumin (BSA) and 1 μL of JumpStart RedTaq (Sigma). Alternatively, some PCR amplifications were performed in 25 μL total volume containing 2 μL of DNA template, 8.5 μL of sterile water, 1 μL of each 10 μM forward and reverse primer, and 12.5 μL of GoTaq^®^ Green Master Mix (Promega). The thermal cycling profile used for PEPCK, NaK, and H3 started with an initial denaturing step of 1 minute at 94°C, then a repeating 30–40 cycles of 30 seconds at 94°C, an annealing step of 0.5–1 minutes at 48°-62°C (depending on primer set and species), 1 min at 72°C, and a final extension of 7 min at 72°C. PCR products were purified and sequenced by Beckman Coulter Genomics (Danvers, MA, USA). Post reaction dye terminator removal was performed using Agencourt CleanSEQ, after which amplicons were sequenced in both directions using an Applied Biosystems PRISM 3730xl DNA Analyzer. Sequencher 5.0.1 was used to edit raw sequences. Ambiguities, presumably due to heterozygosity, were scored as such using IUPAC nomenclature, and missing data was coded missing.

Sequences were aligned using Multiple Sequence Alignment by Log Expectation (MUSCLE) [90]. Aligned gene sequences had the following maximal lengths: H3: 328 bp, NAK: 582 bp, PEPCK: 570 bp. The final molecular matrix thus included 1,480 characters. No insertions, deletions, or significant ambiguities were detected in the final alignments.

### Taxon ages

To assign dates to fossil taxa we used published geochronological data (S6 Table). The median value of the narrowest age interval assigned to each fossil in the literature is used to indicate the first appearance datum in Fig 1 (black dots). Ranges of living species in Fig 1 are limited to the Recent.

### Phylogenetic methods

We used PAUP* [91] and TNT [92] for parsimony tree searches. In PAUP* multistate taxa were treated as uncertain and branch collapse was set to “min”. We used the heuristic search option with random taxon addition sequences (100,000 replicates) and TBR branch swapping. In TNT we also performed New Technology (NT) searches with a combination of sectorial searches (RSS and CSS), 100 iterations of ratchet, 100 rounds of tree fusing, and 100 cycles of tree drifting, setting the search to reach the minimum length 50 times, and then applying a TBR search to the trees produced by the NT search. We rooted all trees with *Euphasia superba*. Ancestral state reconstructions were run in PAUP*. All clade synapomorphies reported here are unambiguous unless noted otherwise (S2 and S3 Tables). Mesquite [93] was used to visualize individual character optimizations. We determined the minimum ages of clades using ghost lineage analysis [17]. Ghost lineages (range extensions that are informed by tree topology) are indicated as cones along branches in Fig 1. The minimum divergence ages for lineages and clades are derived directly from the oldest fossil in each lineage or clade, or by the minimum age of its sister taxon (S6 Table).

The combined dataset was also examined using Bayesian tree-building methods. The Markov k [94] model with equal state frequencies, combined with gamma-distributed rates across sites was applied to the phenomic partition. The “coding = variable” parameter was used to implement a likelihood conditional on character variability. The molecular dataset was partitioned by gene and the best-fit model for each gene partition was applied as defined by jModeltest2 [95] using the three-substitution scheme and Akaike Information Criterion (AIC) [96]. Models were as follows: GTR+G for H3; SYM+I+G for NAK; and HKY+I+G for PEPCK. Two independent runs were performed (each consisting of 4 chains). The analysis ran for 100,000,000 iterations. Convergence was confirmed by examining the potential scale reduction factor and the standard deviation of split frequencies, which reached 1% after about 10,000,000 generations. The first 25% of sampled trees were discarded as burn in. For all analyses we rooted the tree with *Euphasia superba*. All analyses were run using MrBayes v3.1.2b4 [97] implemented in the CIPRES portal [98].

### Tree support and stability

Bremer Support (BS) [99] was calculated in TNT using the script and parameters from [100]. Jackknife analyses was run in TNT with 1,000 re-samplings and percentage deletion of 36% following the parameters discussed in [101]. To account for reductions in support and stability that may be due to the inclusion of fossils with substantial amounts of missing data (S5 Table), we calculated and report BS and jackknife values with and without fossils (Fig 1, values in parenthesis calculated with fossils excluded).

### Definition of industrial-scale aquaculture and farmed species

Worldwide shrimp aquaculture production statistics were analyzed by species to establish the phylogenetic scope of global shrimp farming (S4 Table). The data show that the selection of species that are the subject of active farming has narrowed significantly since the inception of shrimp aquaculture. In 1970 the giant tiger shrimp *Penaeus monodon* was the main aquaculture species, accounting for 24% of total production, but the largest share of production (61%) came from an undetermined number of other species from the clade Agripenaeina ([11], known collectively as the genus *Penaeus* before the taxonomic revision of Perez-Farfante and Kensley [61]). Importantly, at that time, species of *Metapenaeus* (clade Trachypenaeini in Fig 1) accounted for a significant 14% of total shrimp aquaculture. By 1990, 95% of all farmed shrimp came from only three species of Agripenaeina (which contributed 43%, 27%, and 13% of total production respectively), while *Metapenaeus spp*. accounted for only 4% of all farmed shrimp [11]. In 2013, 98% of farmed shrimp belonged to clade Agripenaeina, and there were only two dominant species (contributing 70% and 21% of total production respectively [11]). The contribution of *Metapenaeus spp*. in 2013 was less than 2% of total production. Farming of species of *Metapenaus* has been reduced to low-management systems, using wild animals that enter ponds, and, as such, these farms represent a low productivity, non-industrial type of farming (e.g., [102,103]). Therefore, even though species of *Metapenaeus* are the subject of some farming, and are of local significance in some parts of the world, we exclude them from our definition of industrial-scale global farming.

We also note here that farming of giant freshwater shrimp (genus *Macrobrachium*, Decapoda, Caridea) has experienced significant growth, and in 2013 represented a total value equivalent to 15% of the value generated by farmed marine shrimp [11]. These palaemonids are freshwater shrimp that are not members of the marine clade Penaeoidea, but are instead distant relatives [104]. Evolutionary events that led to large body sizes in these freshwater palaemoid shrimp are convergent relative to the emergence of large size in the marine clade Penaeoidea.

### Clade names

Clades found in this study that have not been named in previous work were given names for the purpose of facilitating the discussion of our phylogenetic analyses. Revisions to these names are possible in the future if these phylogenetic groups are formally incorporated to Penaeoidean nomenclature. We followed the guidelines of de Queiroz [105] for crown clade and total clade definitions. We preserve commonly used nomenclature as much as possible, such that commonly accepted groups (e.g., taxonomic families from the work of Perez-Farfante and Kensley [61]) that are also supported phylogenetically, are simply updated based on phylogenetic results. The new clade Phorcysida is named here in reference to Phorcys, an ancient sea deity in Greek mythology who fathered several sea monsters [106]. The clade includes the current taxonomic families Aristeidae, Benthesicymidae, and Solenoceridae (each of which is also supported as a clade), which are known to inhabit very deep, bathyal, and in some instances, abyssal waters (S1 Fig). This group has previously been recognized on the basis of morphological [10,66] and molecular data [9]. Podobranchida is a crown clade within Phorcysida that includes Aristeidae and Benthesicymidae only. Comparative anatomists have long recognized the close morphological affinities of Aristeidae and Benthesycimidae, and have previously considered them divisions of a single family or sub-family [10,66]. The etymology of the clade name Podobranchida derives from the widespread presence of podobranchia (gills attached to the coxa) in several thoracic segments, a derived characteristic of this clade (S2 Table). The crown clade Agripenaeina is erected here to identify a clade that appears in prior molecular studies [107] as well as in our combined data analysis. Agripenaeina contains all shrimp species historically used in aquaculture and its name references the use of these species for industrial-scale farming. The total clade Pan-Agripenaeina includes crown clade Agripenaeina and its extinct stem taxon ^†^*Acanthochirana smithwoodwardi* to the exclusion of other living members of Penaeini.

The genus *Sicyonia* is also a clade, recognized taxonomically as its own family, Sicyoniidae, separate from Penaeidae [61]. Because Sicyoniidae is equivalent in species content to the genus *Sicyonia*, we provide a phylogenetic definition for *Sicyonia* only (S1 Table). The total clade Pan-*Sicyonia* consists of the crown clade *Sicyonia* and its fossil sister taxon, ^†^*Drobna deformis*. We redefine Penaeidae (S1 Table) based on the phylogenetic results of our study and of Ma *et al*. [9] to include *Sicyonia* as well as all other currently accepted living members of the taxonomic family Penaeidae [61].

## Extended Results and Optimizations

### Combined data parsimony-extended results

Of the 339 phenomic characters in our matrix, 47 were invariant, 37 variable characters were uninformative, and 255 variable characters were parsimony-informative. Of 1,480 molecular characters 841 were invariant, 109 were not informative, and 530 were parsimony-informative. Twenty-four most parsimonious trees were found, 4272 steps long, with overall consistency index = 0.317, retention index = 0.591, and homoplasy index = 0.683. Fig 1 shows the topology of the strict consensus tree from this analysis, including BS and jackknife values.

We conducted sensitivity analyses on the tree topology under the exclusion of fossil taxa and of three characters that are key to our main conclusions. The tree that resulted from the exclusion of fossil taxa from parsimony searches was topologically congruent with the tree that included fossils shown in Fig 1. Bremer support and jackknife values were higher when fossils were excluded and we report both sets of values (with and without fossils) in Fig 1. Exclusion of water depth preference and temperature preference (either alone or in combination) from the combined data matrix also resulted in a tree that was congruent with the tree shown in Fig 1. Thus, inclusion of fossils or of behavioral characters did not affect tree topology. Exclusion of body size changed the position of the fossil ^†^*Acanthochirana smithwoodwardi* to inside the clade Agripenaeina, but had no topological effects in other areas of the tree.

In the combined data tree, Penaeoidea is a well-supported clade (BS = 2, BS_no fossils_ = 15) and our analysis supports its sister relationship with Sergestoidea. Penaeoidea split into two clades, one of which includes species with a deep-water preference, while the other includes species with a shallow-water preference (for bathymetry optimization see S1 Fig). Four out of the five family-level groupings recognized by traditional taxonomy [61] are also clades in the combined data tree, namely Aristeidae, Benthesicymidae, Solenoceridae, and Sicyoniidae (= *Sicyonia*). The fifth taxonomic family, Penaeidae as defined by Perez-Farfante and Kensley [61] would be paraphyletic, because the phylogenetic results show that *Sicyonia* is nested within it. Thus, Penaeidae is redefined here phylogenetically, and encompasses all the shallow water species, including the clade *Sicyonia*. Support for Penaeidae is low (BS = 1, BS_no fossils_ = 2). Phenomic synapomorphies for the clade Penaeidae are listed in S2 Table, and include the presence of an adrostral carina (character 14), a cervical sulcus that is short (character 39), and several thoracic epipods with a bifurcated shape (characters 89, 129, and 153).

The split of Penaeidae into three major clades, Penaeini, Parapenaeini, and Trachypenaeini, which was proposed by Burkenroad based on morphology [10], is supported in our combined data tree and in previous molecular phylogenies [9]. In contrast to Burkenroad’s arrangement [10], however, phylogenetic analyses place *Sicyonia* inside Trachypenaeini, and we redefine Trachypenaeini accordingly (S1 Table). The clade Trachypenaeini has overall low support (BS = 1, BS_no fossils_ = 2). Synapomorphies of this clade include the lack of a pleurobranchia in the penultimate thoracic segment (character 193), the presence of a post-ocular sulcus (character 55), and a last thoracic leg conspicuously longer than the penultimate thoracic leg (character 218). The Trachypenaeini are predominantly sub-littoral, live in warm waters (S1 and S3 Figs), and include species of importance to fisheries, as well as the genus *Metapenaeus*, the only species outside of clade Agripenaeina that has been the subject of some farming (see Materials and Methods). Parapenaeini, the sister clade to Trachypenaeini, was originally erected based on the presence of fixed spines on the telson, and of a ventromesial spine on the first article of the antennular peduncle [10]. Our combined data phylogeny recovers this clade with moderate support (BS = 1, BS_no fossils_ = 6) with six unambiguous synapomorphies (S2 Table): presence of a pterygostomian spine (character 62), a dorsal spine on abdominal segments 4 (character 244) and 5 (character 245), the first 2 abdominal segments lacking a notch at the level of the hinge (characters 271 and 273), and fixed spines on the telson (character 309). Parapenaeini also includes species of significance to fisheries, particularly members of *Parapenaeus* and *Metapenaeopsis* [51].

The third penaeid clade, Penaeini, includes species that have been well-studied in terms of their reproduction, physiology, and immune defenses, because they are significant to fisheries and to farming (specifically, the members of Agripenaeina). Two unambiguous synapomorphies of this clade are an articulated palp on the first maxilla (character 73) and a bifurcated epipod associated with the third walking leg (pereiopod 3, character 174). Combined data support for clade Penaeini is relatively high (BS = 2, BS_no fossils_ = 19). Within Penaeini, the genera with species that have been the subject of industrial-scale farming form a clade with moderate support (BS = 1, BS_no fossils_ = 4), and it is phylogenetically defined and named in this study as clade Agripenaeina (S1 Table). Members of Agripenaeina share a derived character of very large body size (character 316, S2 Table). Because Agripenaeina has a fossil stem taxon, ^†^*Acanthochirana smithwoodwardi* (Fig 1), and many characters are not known for this species (80% missing data, S2 Table), a number of derived characters are technically ambiguous synapomorphies of Agripenaeina, until more data allows us to determine whether to assign transformations to the total clade or to the crown clade node. Thus, these characters are either synapomorphies of Agripenaeina or of Pan-Agripenaeina, and include an adrostral sulcus present (character 16), anterolateral carina present (character 48), uniformly wide pleopods (character 282), and a closed thelycum (character 299). These additional synapomorphies of Agripenaeina are listed in S3 Table.

The deep-water clade (Phorcysida) includes three traditional taxonomic families that are now recognized as clades both here and in prior molecular work [9]: Solenoceridae, Benthesicymidae, and Aristeidae. Aristeidae and Benthesicymidae are sister clades and capture most penaeoidean species with very deep-water behaviors (exceeding 4,000 m in depth, S1 Fig). Aristeidae and Benthesicymidae (clade Podobranchida) share numerous derived characters, such as a rounded orbital margin (character 53), a second maxilla with a palp lacking spinules (character 77), podobranchiae in the 9^th^ through 13^th^ thoracic segments (characters 109, 137, 161, and 182), very conspicuous hinges between the 1^st^ and 2^nd^ and between the 2^nd^ and 3^rd^ segments of the abdomen (character 269), and several other synapomorphies listed in S2 Table. Phylogenetic support for Podobranchida is high (BS = 3, BS_no fossils_ = 16).

Solenoceridae is the third major clade in the deep-water group, and is recovered with low support in the combined data phylogeny (BS = 1, BS_no fossils_ = 2). Synapomorphies of Solenoceridae include the presence of a post-antennal spine (character 60), a conspicuously short exopod in the third maxilliped (character 100), a short 6^th^ abdominal segment (i.e., it is not the longest segment, character 247), a distolateral “spur” in the second swimming leg of males (character 298), and fixed spines in the telson (character 309, S2 Table).

### Fossil placements and minimum clade ages

As described in Materials and Methods, we limited fossil sampling based on our ability to access specimens, completeness and preservation of specimens, and potential significance of each fossil to the evolution of the clade. The oldest putative penaeoid, ^†^*Aciculopoda mapesi* [108] of Devonian age, was available for study (USNM 540766), but it is our assessment that poor preservation precludes scoring of most characters for this taxon (98.5% missing data). Therefore, contra [108] we find insufficient evidence at present to support the assignment of ^†^*A*. *mapesi* to the clade Penaeoidea, based on the very few features preserved in the single known specimen. The next earliest Period with a record of putative penaeoids is the Triassic. ^†^*Ifasya madagascariensis* from the Early Triassic of Madagascar [109,110] has traditionally been assigned to the taxonomic family Penaeidae [60]. Our study corroborates the assignment of ^†^*I*. *madagascariensis* to Penaeidae, and specifically places it within Parapenaeini (Fig 1). This placement of the Early Triassic ^†^*Ifasya* implies that the lineages that gave rise to the three major penaeid clades (Penaeini, Parapenaeini, and Trachypenaeini) were already distinct by the Early Triassic.

Two synapomorphies link ^†^*I*. *madagascariensis* and ^†^*Aeger tipularius* in our analysis: the absence of dorsal rostral teeth and the presence of a ventral rostral tooth (characters 3 and 5, respectively). ^†^*Aeger tipularius* in turn shares with the extant *Artemesia longinaris* features that are not known for ^†^*I*. *madagascariensis*: very long antennular flagella (characters 222 and 224) and very long, roughly flagelliform pleopods (characters 282 and 283). These features are more common in the deep-water penaeoids than in Penaeidae, but are synapomorphies for the *Artemesia* plus ^†^*Aeger tipularius* clade, based on our tree. We interpret this possible affinity between ^†^*A*. *tipularius* and *A*. *longinaris* cautiously because carapace ornamentations were not preserved in the ^†^*A*. *tipularius* specimens studied by us, and most of these characters are scored as missing data in our matrix. We also note that Bayesian methods find an alternative placement for ^†^*A*. *tipularius* (S4 Fig and discussion below), indicating an unstable position of this fossil within Parapenaeini. We emphasize, however, that our assessment of the Early Triassic minimum age of Penaeidae does not depend upon the position of ^†^*Aeger*. Taxonomic (i.e., non-phylogenetic) arrangements have grouped ^†^*Aeger* with other fossil species that possess a spinose third maxilliped [60].

Derived characters place ^†^*Drobna deformis* as the stem taxon to crown *Sicyonia*, including an unusually elevated and short carapace (character 66), spinose projections on the pleura of several abdominal segments (characters 249, 251, and 252), and a strong narrowing of the cuticle covering the dorsal portion of the first abdominal segment (character 279). Sicyonians display external features that distinguish them conspicuously from other penaeoids (see [61]). Based on our examination of ^†^*D*. *deformis*, the lineage that gave rise to *Sicyonia* started to diverge morphologically from all other Trachypenaeini at least by the Late Jurassic (Fig 1). This split involved the re-configuration of the carapace and of the abdominal cuticle, as shown by the synapomorphies that link ^†^*Drobna* with *Sicyonia* (listed above and more completely in S3 Table). The Late Jurassic minimum age of Trachypenaeini indicates a time of origin for the clade that predates the fossil record of modern mangrove forests [16]. Like species in Agripenaeina, living members of Trachypenaeini (e.g., *Metapenaeus*) are strongly associated with mangrove forests as juveniles [6,103].

The placement of ^†^*Antrimpos speciosus* inside clade Agripenaeina is a key finding of our study. We note here that other fossils have been assigned (non-phylogenetically) to the genus ^†^*Antrimpos*, including fossils that predate ^†^*A*. *speciosus* (e.g. [20,111]) but sufficiently preserved specimens of these species were not available to us for direct phylogenetic study. Furthermore, attributions of fossils to the genus ^†^*Antrimpos* have often been weakly justified, and previous authors have recognized the need to reexamine this taxon, which has been termed a wastebasket taxon (see [110,112] and references therein). For example, ^†^*Ifasya madagascariensis* (included in our study) was originally described as a member of the genus ^†^*Antrimpos* [109], but was recently re-named based on close examination of well-preserved specimens [110].

The genera ^†^*Aeger*, ^†^*Acanthochirana*, ^†^*Anisaeger*, and ^†^*Distaeger* are taxonomically grouped into family ^†^Aegeridae [113]. This group has extinct members only, and a stratigraphic range that goes back into the Middle Triassic [60,114]. ^†^Aegeridae accommodates shrimp with a third maxilliped that is covered with abundant long thin spines (or thick setae). Besides this character, very little obvious morphological affinity can be observed in all assigned members of ^†^Aegeridae, and paleontologists have acknowledged the need to reconsider the status of this family phylogenetically [114]. We note here that our analysis includes two genera from ^†^Aegeridae, for which specimens were available for direct study: ^†^*Aeger* and ^†^*Acanthochirana*. We find no phylogenetic support for ^†^Aegeridae as a clade, based on the inclusion of these two taxa. ^†^*Aeger* places phylogenetically within Parapenaeini (as discussed above), while ^†^*Acanthochirana* belongs to Penaeini. The synapomorphies that link ^†^*Acanthochirana smithwoodwardi* with Agripenaeina (S3 Table) include numerous post-rostral teeth (more than 4 teeth, character 8), absence of a branchiocardiac carina (character 56), and a partitioned cicatrix in the last abdominal segment (characters 261 and 262). Unlike the Agripenaeina, ^†^*A*. *smithwoodwardi* is not known to reach very large body sizes based on the available record [115].

The fossil record attributed to the deep-water penaeoid clade (Phorcysida) is very sparse. Apart from ^†^*Palaeobenthesicymus libanensis* (which is included in this study) only two putative solenocerids, ^†^*Archeosolenocera straeleni* from the Middle Jurassic of France [116], and ^†^*Eogordonella iranianiensis* from the Eocene of Iran [117], have been attributed to this clade. Specimens from these species were not available for direct study. ^†^*Paleomattea deliciosa* from the Lower Cretaceous of Brazil is the oldest fossil currently assigned to Sergestoidea [118]. We corroborate its position in a clade with other sergestoids, and further refine some of its relationships within the group (Fig 1).

### Combined data Bayesian results

The phylogeny of Penaeoidea estimated by Bayesian Inference (50% majority rule consensus tree, S4 Fig) has the same general topology as the parsimony strict consensus tree shown in Fig 1. In general, for nodes in common between the parsimony and Bayesian trees, support is higher in the Bayesian tree than in the parsimony tree, including nodes representing early splits. The tendency of Bayesian posterior probabilities to indicate higher support than jackknife values has been documented previously [101]. The topology supporting our major conclusions, the composition of the major clades, and the relationships among clades are in agreement between the Bayesian and parsimony analyses. As in parsimony analysis, the Bayesian tree shows a basal split of Penaeoidea into deep-water and shallow-water clades. The topology inside each of these two major clades is also similar between analyses, with the shallow-water clade composed of Penaeini as sister to the Parapenaeini + Trachypenaeini clade, and the deep-water clade with Solenoceridae as sister to the Podobranchida clade. Most of the differences between parsimony and Bayesian results are at the level of inter-generic relationships within the extant Aristeidae, Solenoceridae, Trachypenaeini, and Parapenaeini (compare Fig 1 with S3 Fig). Regarding fossil placements found by each method, the positions of ^†^*A*. *speciosus*, ^†^*D*. *deformis*, ^†^*I*. *madagascariensis*, ^†^*P*. *libanensis*, and ^†^*P*. *deliciosa* are the same between the two trees. Only two noteworthy differences are observed. ^†^*A*. *smithwoodwardi* places as stem taxon to the entire clade Penaeini by Bayesian analysis, in contrast to its position as a stem taxon to Agripenaeina in parsimony. If corroborated, this position would not change minimum clade ages for either the deep water or the shallow water clades, or the minimum age of the clade Agripenaeina. Secondly, perhaps the most important difference between Bayesian and parsimony results is the position of ^†^*A*. *tipularius*, which is assigned to Solenoceridae (in the deep-water clade) by the Bayesian analysis. This result, if confirmed by further work, would represent the oldest phylogenetically supported minimum age for Solenoceridae. Neither of these topological differences impacts the major findings of our study regarding the origin of Agripenaeina.

## Supporting Information

S1 Fig Optimization of maximum depth (character 320). One of 24 most parsimonious trees (4272 steps) from the combined data parsimony analysis. The common ancestor of Trachypenaeini and Parapenaeini is reconstructed by optimization as sub-littoral, but the common ancestors of Penaeini and of the entire clade Penaeidae optimize as ambiguous due to the basal positions and deep-water behaviors of *Funchalia* and *Pelagopenaeus*. The common ancestor of the deep-water clade (Phorcysida) is reconstructed by optimization as having being bathyal. The bathymetry characterization of the common ancestor of all Penaeoidea remains ambiguous. All clades show the same optimization pattern in every shortest tree, with the exception of Aristeidae, which is not fully resolved (Fig 1).
(PDF)

S2 Fig Optimization of maximum body size (character 316). One of 24 most parsimonious trees (4272 steps) from the combined data parsimony analysis. Large body size is a derived character of clade Agripenaeina. Members of Aristeidae are also large-bodied, but because this clade is not fully resolved (see consensus tree in Fig 1), three of 24 shortest trees (not shown) are ambiguous regarding the ancestral size of Aristeidae. All other clades show the same optimization pattern in every shortest tree.
(PDF)

S3 Fig Optimization of minimum water temperature inhabited by each taxon (character 322). One of 24 most parsimonious trees (4272 steps) from the combined data parsimony analysis. The overall distribution of minimum water temperature (character 322) tracks closely with maximum water depth shown in S1 Fig. Agripenaeina is a warm-water clade but the ancestry of Penaeini and of Penaeidae regarding this character is uncertain. Missing temperature data for *Heteropenaeus* contributes to this ambiguity, but we note that based on the shallow-water and tropical to sub-tropical distribution of this species [61], it is likely to be restricted to warm waters. Optimization of this character shows the same pattern in every shortest tree.
(PDF)

S4 Fig Bayesian 50% majority rule consensus tree of combined data. Values to the right of each node are posterior probabilities. Membership in all major clades is congruent with the parsimony tree (Fig 1), with the exception of ^†^*Aeger tipularius*, which places within Solenoceridae in this tree, but belongs to Parapenaeini based on the parsimony trees. The position of ^†^*Acanthochirana smithwoodwardii* relative to other members of clade Penaeini also differs between this tree and the parsimony consensus (see Fig 1).
(PDF)

S1 Table Major clades from combined data parsimony analysis. (PDF)

S2 Table Synapomorphies of major clades in this study based on shortest parsimony trees. (PDF)

S3 Table Additional synapomorphies of crown clades with extinct sister taxa. (PDF)

S4 Table Global shrimp aquaculture production by species based on Food and Agriculture Organization of the United Nations (FAO) statistics (http://www.fao.org/fishery/statistics/en). Italicized values are FAO estimates, and some zero (0) values may correspond to very small contributions, rounded to 0. Species names are as reported by FAO, with those that belong to Agripenaeina indicated in bold.
(PDF)

S5 Table Taxa sampled in the study and summary of data collected. (PDF)

S6 Table Age information and number of specimens sampled for fossil taxa in this study. (PDF)
