# Supplementary figures and images for: The Origin of Large-Bodied Shrimp that Dominate Modern Global Aquaculture
Source: PLoS One. 2016 Jul 14;11(7):e0158840. doi: 10.1371/journal.pone.0158840 (PMC4945062; doi:10.1371/journal.pone.0158840)

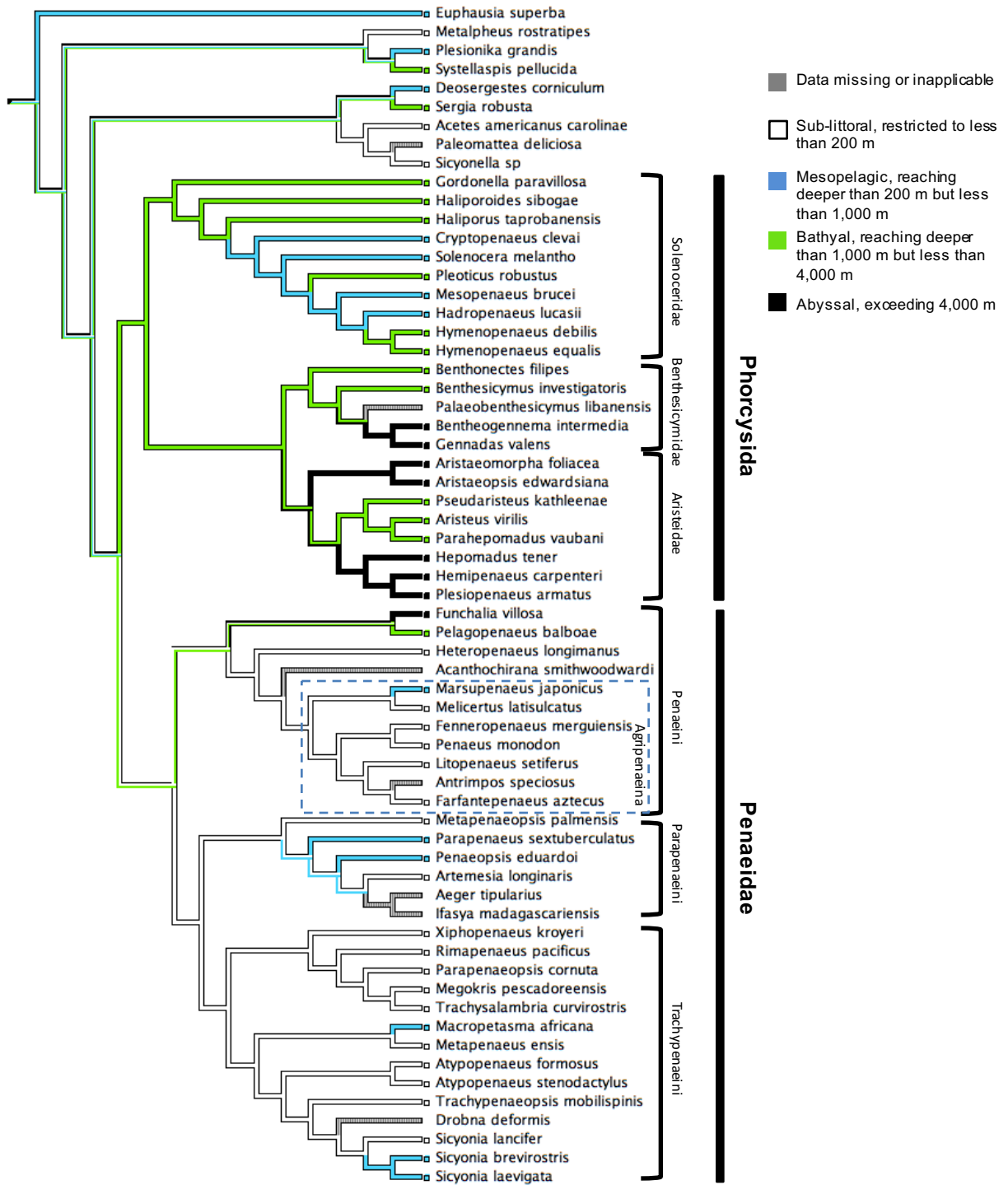

Supplement: S1 Fig — One of 24 most parsimonious trees (4272 steps) from the combined data parsimony analysis. The common ancestor of Trachypenaeini and Parapenaeini is reconstructed by optimization as sub-littoral, but the common ancestors of Penaeini and of the entire clade Penaeidae optimize as ambiguous due to the basal positions and deep-water behaviors of Funchalia and Pelagopenaeus. The common ancestor of the deep-water clade (Phorcysida) is reconstructed by optimization as having being bathyal. The bathymetry characterization of the common ancestor of all Penaeoidea remains ambiguous. All clades show the same optimization pattern in every shortest tree, with the exception of Aristeidae, which is not fully resolved (Fig 1). (PDF) [file pone.0158840.s001.pdf]

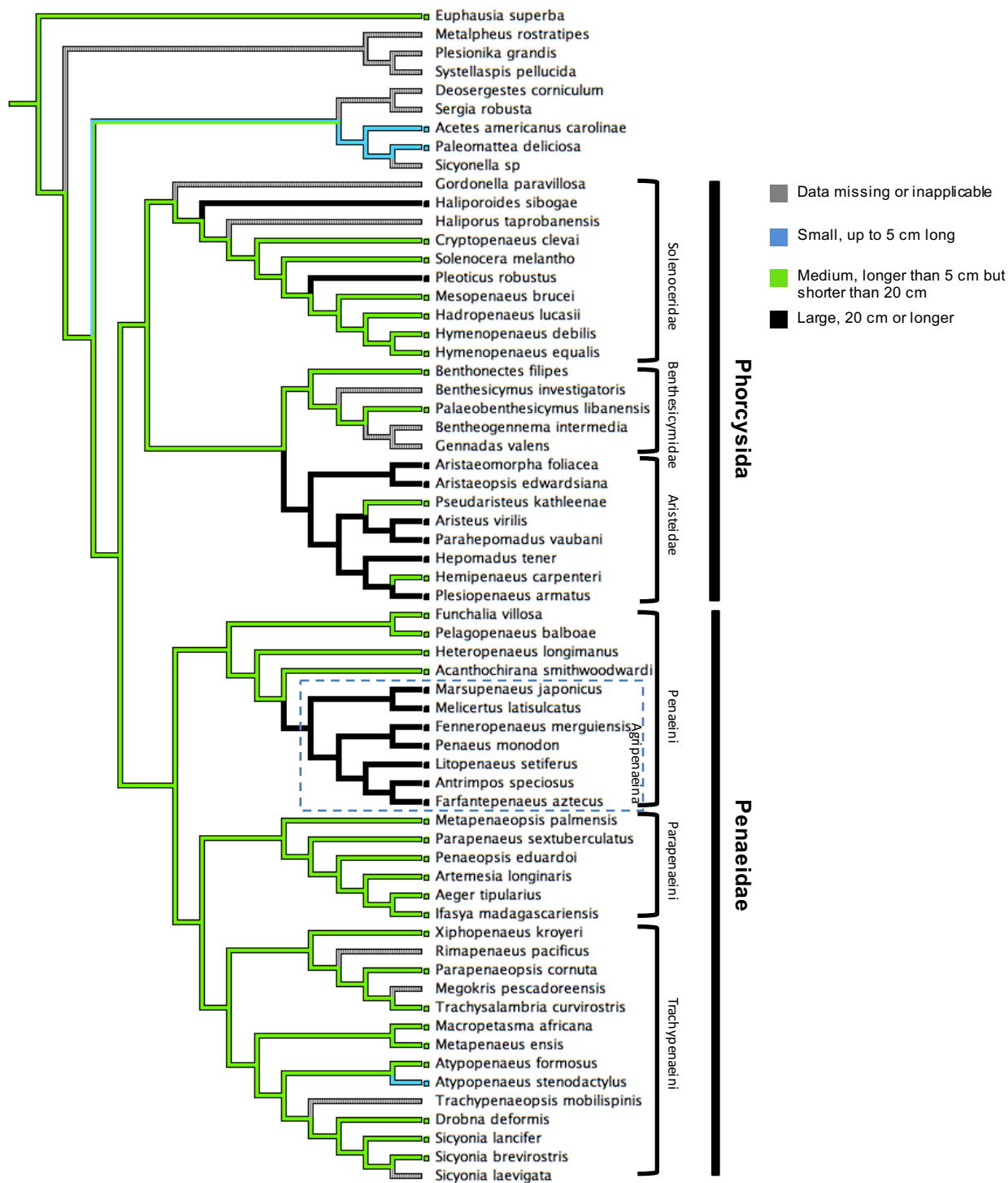

Supplement: S2 Fig — One of 24 most parsimonious trees (4272 steps) from the combined data parsimony analysis. Large body size is a derived character of clade Agripenaeina. Members of Aristeidae are also large-bodied, but because this clade is not fully resolved (see consensus tree in Fig 1), three of 24 shortest trees (not shown) are ambiguous regarding the ancestral size of Aristeidae. All other clades show the same optimization pattern in every shortest tree. (PDF) [file pone.0158840.s002.pdf]

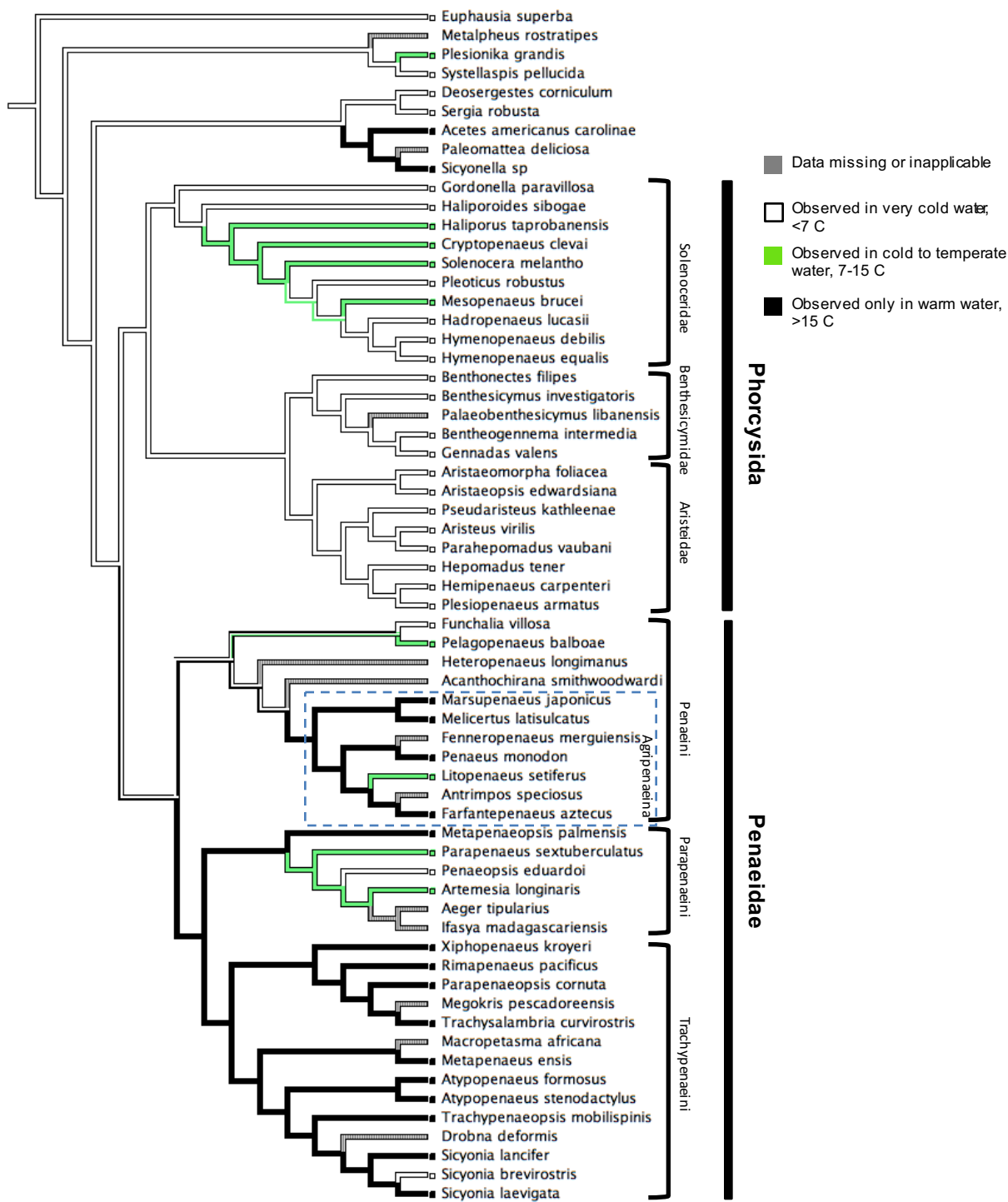

Supplement: S3 Fig — One of 24 most parsimonious trees (4272 steps) from the combined data parsimony analysis. The overall distribution of minimum water temperature (character 322) tracks closely with maximum water depth shown in S1 Fig. Agripenaeina is a warm-water clade but the ancestry of Penaeini and of Penaeidae regarding this character is uncertain. Missing temperature data for Heteropenaeus contributes to this ambiguity, but we note that based on the shallow-water and tropical to sub-tropical distribution of this species [61], it is likely to be restricted to warm waters. Optimization of this character shows the same pattern in every shortest tree. (PDF) [file pone.0158840.s003.pdf]

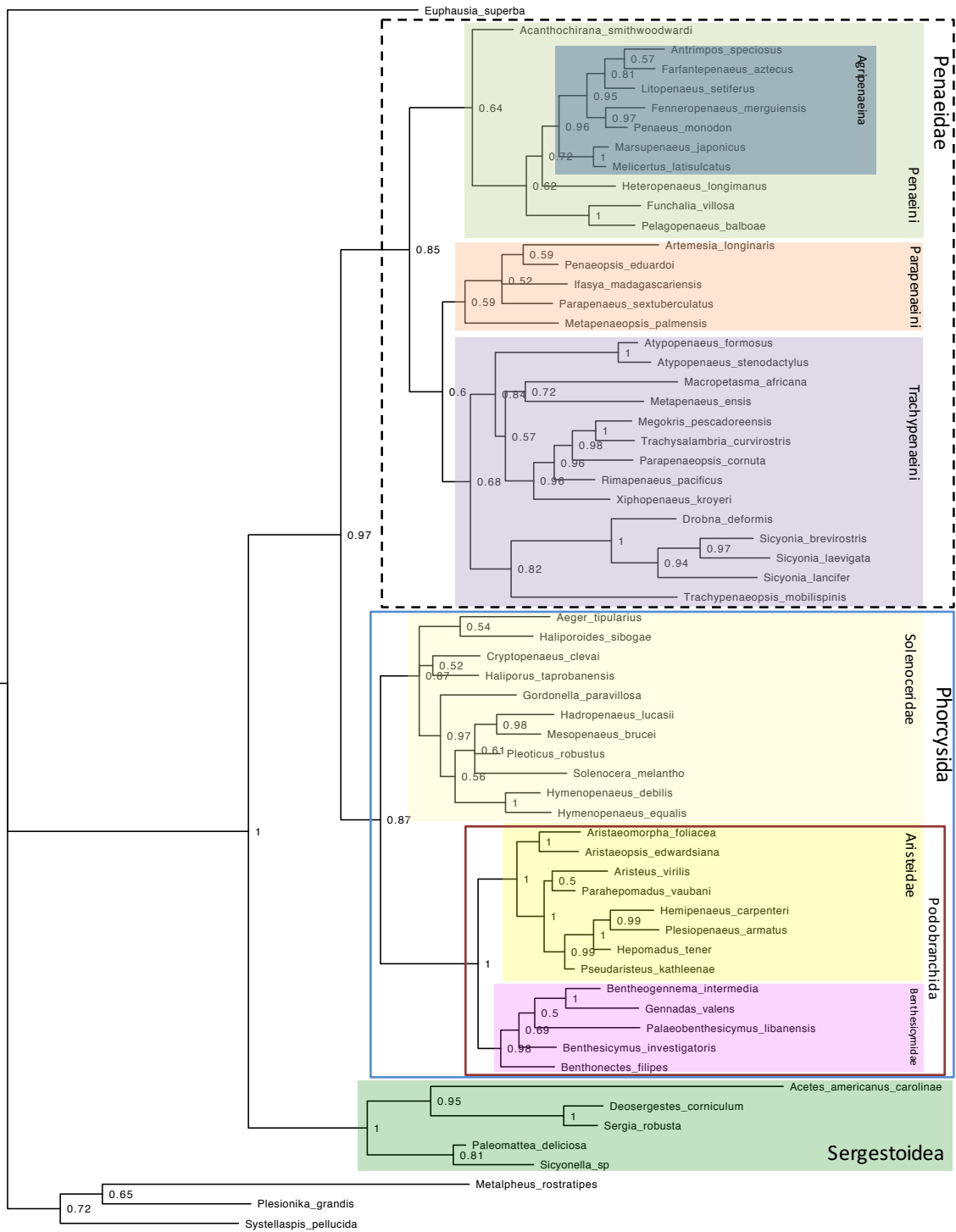

Supplement: S4 Fig — Values to the right of each node are posterior probabilities. Membership in all major clades is congruent with the parsimony tree (Fig 1), with the exception of †Aeger tipularius, which places within Solenoceridae in this tree, but belongs to Parapenaeini based on the parsimony trees. The position of †Acanthochirana smithwoodwardii relative to other members of clade Penaeini also differs between this tree and the parsimony consensus (see Fig 1). (PDF) [file pone.0158840.s004.pdf]
