# Supplementary material for: The Origin of Large-Bodied Shrimp that Dominate Modern Global Aquaculture
Source: PLoS One. 2016 Jul 14;11(7):e0158840. doi: 10.1371/journal.pone.0158840 (PMC4945062; doi:10.1371/journal.pone.0158840)
Supplement: S1 Table — (PDF) [file pone.0158840.s005.pdf]

S1 Table. Major clades from combined data parsimony analysis.

Clade	Definition
Penaeoidea	The common ancestor of *Aristaeomorpha foliacea* and *Penaeus monodon* and all of its descendants
Penaeidae	The common ancestor of *Penaeus monodon* and *Atypopenaeus formosus* and all of its descendants
Penaeini	The common ancestor of *Penaeus monodon* and *Pelagopenaeus balboae* and all of its descendants
Pan-Agripenaeina	All species more closely related to Agripenaeini than to *Heteropenaeus longimanus*
Agripenaeina	The common ancestor of *Penaeus monodon* and *Marsupenaeus japonicus* and all of its descendants
Parapenaeini	The common ancestor of *Metapenaeopsis palmensis* and *Artemesia longinaris* and all of its descendants
Trachypenaeini	The common ancestor of *Xiphopenaeus kroyeri* and *Atypopenaeus formosus* and all of its descendants
Pan-Sicyonia	All species more closely related to crown clade Sicynonia that to *Trachypenaeopsis mobilispinis*
Sicyonia	The common ancestor of *Sicyonia lancifer* and *Sicyonia laevigata* and all of its descendants
Phorcysida	The common ancestor of *Aristaeomorpha foliacea* and *Hymenopenaeus equalis* and all of its descendants
Podobranchida	The common ancestor of *Aristaeomorpha foliacea* and *Bentheogennema intermedia* and all of its descendants
Aristeidae	The common ancestor of *Aristaeomorpha foliacea* and *Pseudaristeus kathleenae* and all all of its descendants
Benthesicymidae	The common ancestor of *Benthonectes filipes* and *Gennadas valens* and all of its descendants
Solenoceridae	The common ancestor of *Gordonella paravillosa* and *Hymenopenaeus equalis* and all of its descendants
Sergestoidea	The common ancestor of *Sergia robusta* and *Acetes americanus carolinae* and all of its descendants
