# Supplementary material for: The Origin of Large-Bodied Shrimp that Dominate Modern Global Aquaculture
Source: PLoS One. 2016 Jul 14;11(7):e0158840. doi: 10.1371/journal.pone.0158840 (PMC4945062; doi:10.1371/journal.pone.0158840)
Supplement: S2 Table — (PDF) [file pone.0158840.s006.pdf]

S2 Table. Synapomorphies of major clades in this study based on shortest parsimony trees

Clade	Unambiguous synapomorphies	MorphoBank character numbers
Penaeoidea	dorsal rostral teeth present, post-rostral carina long, hepatic sulcus present, orbito-antennal sulcus present, antennal spine present, arthrobranchia in maxilliped 1 present, anterior arthrobranchia in maxilliped 2 present, maxilliped 3 pleurobranchia present, exopod on pereiopod 4 present, exopod on pereiopod 5 present, disto-lateral spine on outer margin of first antennular peduncle present, dorsoabdominal carinae on somites 4, 5, and 6 (3 characters), dorsoposterior spine on abdominal somite 6, longitudinal carinae on endopod of uropod, telson with lateral movable spines, more than 4 pairs of dorsolateral movable spines in telson (if present), sperm with acrosome (data missing in all sampled Caridea)	3, 19, 26, 49, 58, 84, 92, 103, 188, 203, 227, 237, 238, 239, 246, 305, 311, 314, 324
Penaeidae	last dorsal tooth (epigastric) distinctively separated from other dorsal teeth (applicable only if more than 1 tooth is present behind the rostrum), adrostral carina present, short cervical sulcus, distal article of manidular palp larger than proximal article, spinules on palp of maxilla 1, epipod of second maxilliped bifurcated, pereiopod 1 with basal and ischial spines (2 characters), epipod of pereiopod 1 bifurcated, epipod of pereiopod 2 bifurcated, pereiopod 3 longer than pereiopod 2 by at least 30%, antennular prosartema present (conspicuously), ocular scale present, trumpet shaped appendix masculina	9, 14, 39, 71, 74, 89, 111, 112, 129, 153, 213, 219, 288, 297
Penaeini	multiarticulated palp of maxilla 1, bifurcate epipod on pereiopod 3	73, 174
Pan-Agripenaeina	4 or more post-rostral teeth, branchiocardiac carina absent, cicatrix on somite 6 present	8, 56, 261
Agripenaeina	large body size	316
Parapenaeini	pterygostomian spine present, dorsoposterior spine on abdominal somites 4 and 5 (2 characters), pleurae of somites 1 and 2 lacking notches at hinge site (2 characters), fixed spines on telson present	62, 244, 245, 271, 273, 309
Trachypenaeini	post-ocular sulcus present, pleurobranchia on pereiopod 4 segment lacking, pereiopod 5 longer than pereiopod 4 (by more than 30%)	55, 193, 218
Pan-Sicyonia	roughly square carapace, pereiopod 3 longer than pereiopod 4 (by more than 30%), spines on pleural margins of at least somites 2, 4, and 5 (3 characters), narrow dorsal cuticle on abdominal somite 1	66, 216, 249, 251, 252, 279
Sicyonia	apical teeth present, anterodorsal projection on abdominal somite 1, deep traverse sulci on abominal segments, anterior hinges (between somites 1-2 and 2-3) completely inconspicuous (hidden)	2, 257, 268, 269
Phorcysida	Branchiocardiac sulcus present, exopods on all pereiopods small (5 characters), ventral antennular flagellum longer than carapace, dorsoposterior spine on abdominal somite 5 present, long pleopods (2.5 times the protopod or longer), ocular tubercle present, male appendix interna present	57, 127, 151, 172, 189, 204, 222, 245, 283, 287, 294
Podobranchida	branchiostegal and hepatic carinae fused, continuously rounded orbital margin, palp of maxilla 2 lacking spinules, arthrobranchia on maxilliped 1 well developed, anterior arthrobranchia on second maxilliped small, pleurobranchia on maxilliped 3 small, podobranchia on maxilliped 3 present, pleurobranchia on pereiopod 1 small, podobranchiae on pereiopods 1-3 (3 characters), pereiopod 4 is longer than pereiopod 3 (by at least 30%), anterior abdominal hinges (between somites 1-2 and 2-3) very conspicuous, raised double notches on pleurae of somites 1 and 2 involved in abdominal hinges (2 characters)	35, 53, 77, 85, 93, 103, 109, 132, 137, 161, 182, 216, 269, 272, 274
Aristeidae	adrostral carina present, dactyl of maxilliped 3 with strong proximal depression, pleurobranchiae on pereiopods 2-4 small (3 characters), cicatrix on abdominal somite 6, large body size, abyssopelagic maximal depth	14, 110, 156, 177, 194, 261, 316, 320
Benthesicymidae	antennal spine absent, third article of antennular peduncle long (as long or longer than second article), very small cornea of the eye	58, 232, 291
Solenoceridae	post-antennal spine present, exopod of maxilliped 3 short, abdominal somite 6 not the longest somite, distolateral projection (spur) on male second pleopod, telson with fixed spines	60, 100, 247, 298, 309
Sergestoidea	rostrum not reaching cornea anteriorly, posterior submarginal carina absent, maxilliped 2 exopod absent, maxilliped 3 exopod absent, pleurobranchia absent in all pereiopods (5 characters)	1, 65, 86, 99, 131, 155, 176, 193, 208
