# Supplementary material for: The Origin of Large-Bodied Shrimp that Dominate Modern Global Aquaculture
Source: PLoS One. 2016 Jul 14;11(7):e0158840. doi: 10.1371/journal.pone.0158840 (PMC4945062; doi:10.1371/journal.pone.0158840)
Supplement: S3 Table — (PDF) [file pone.0158840.s007.pdf]

S3 Table. Additional synapomorphies of crown clades with extinct sister taxa*

Clade	Additional synapomorphies*	MorphoBank character numbers
Agripenaeina	adrostral sulcus present, anterolateral carina present, uniformly wide pleopods, closed thelycum	16, 48, 282, 299
Sicyonia	adrostral carina absent, cervical sulcus absent, orbital spine absent, continuously rounded orbital margin, maxilla 2 lacking spinules on palp, maxilla 2 with unilobed proximal endite, maxilliped 1 with endopod shorter than exopod, maxilliped 2 lacking an exopod, maxilliped 3 lacking an exopod, all pereiopods lacking an exopod (5 characters), pleurobranchiae lacking in segments bearing pereiopods 1-4 (4 characters), carpal-propodal grooming brushes lacking in females, epipod on pereiopod 3 bifurcated, antennular prosartema not conspicuous, dorsoabdominal carina on somite 1 present, dorsoabdominal carina on somite 2 present, uniramous pleopods (lacking an endopod), ocular stylet present, ocular scale absent, appendix masculina shaped as trumpet with membranous projection	14, 38, 51, 53, 77, 78, 82, 86, 99, 126, 150, 171, 188, 201, 131, 155, 176, 193, 139, 174, 219, 234, 235, 281, 286, 288, 297

* Synapomorphies that technically optimize ambiguously to crown clades with extinct sister taxa. These synapomorphic changes happened either at the crown clade or the total clade node. For clade definitions see S1 Table.
