# Supplementary material for: The Origin of Large-Bodied Shrimp that Dominate Modern Global Aquaculture
Source: PLoS One. 2016 Jul 14;11(7):e0158840. doi: 10.1371/journal.pone.0158840 (PMC4945062; doi:10.1371/journal.pone.0158840)
Supplement: S4 Table — Italicized values are FAO estimates, and some zero (0) values may correspond to very small contributions, rounded to 0. Species names are as reported by FAO, with those that belong to Agripenaeina indicated in bold. (PDF) [file pone.0158840.s008.pdf]

S4 Table. Global shrimp aquaculture production by species based on Food and Agriculture Organization of the United Nations (FAO) statistics (http://www.fao.org/fishery/statistics/en). Italicized values are FAO estimates, and some zero (0) values may correspond to very small contributions, rounded to 0. Species names are as reported by FAO, with those that belong to Agripenaeina indicated in bold.
nei=not included elsewhere

		Aquaculture production per year in tons*					Species contribution (% of year total)				
**Common name**	**Species§**	**1970**	**1980**	**1990**	**2000**	**2013**	**1970**	**1980**	**1990**	**2000**	**2013**
Akiami paste shrimp	*Acetes japonicus*	0	166	1389	544	0	0%	0%	0%	0%	0%
Atlantic ditch shrimp	*Palaemonetes varians*	0	0	0	0	5	0%	0%	0%	0%	0%
**Banana prawn**	***Penaeus merguiensis***	**1623**	**14918**	**32823**	**50393**	**19811**	**18%**	**21%**	**5%**	**4%**	**1%**
**Blue shrimp**	***Penaeus stylirostris***	**0**	***1658***	**8825**	**1824**	**2164**	**0%**	**2%**	**1%**	**0%**	**0%**
**Brown tiger prawn**	***Penaeus esculentus***	**0**	**0**	**7**	**0**	**0**	0%	0%	0%	0%	0%
**Caramote prawn**	***Penaeus kerathurus***	**0**	**0**	**0**	**0**	**0**	0%	0%	0%	0%	0%
Common prawn	*Palaemon serratus*	0	0	0	0	15	0%	0%	0%	0%	0%
Eastern school shrimp	*Metapenaeus macleayi*	0	0	10	0	0	0%	0%	0%	0%	0%
**Fleshy prawn**	***Penaeus chinensis***	**86**	***9820***	**185074**	***193497***	**41973**	**1%**	**14%**	**27%**	**17%**	**1%**
**Giant tiger prawn**	***Penaeus monodon***	***2122***	**16549**	**289774**	**630984**	**803580**	**24%**	**23%**	**43%**	**55%**	**21%**
Greasyback shrimp	*Metapenaeus ensis*	322	684	677	233	155	4%	1%	0%	0%	0%
**Indian white prawn**	***Penaeus indicus***	***10***	***150***	**6736**	**16444**	**23592**	**0%**	**0%**	**1%**	**1%**	**1%**
**Kuruma prawn**	***Penaeus japonicus***	**311**	**1572**	**9417**	**2838**	**47652**	**3%**	**2%**	**1%**	**0%**	**1%**
Metapenaeus shrimps nei	*Metapenaeus spp*	941	8502	28580	20547	63485	10%	12%	4%	2%	2%
Natantian decapods nei	*Natantia*	72	124	430	540	0	1%	0%	0%	0%	0%
Palaemonid shrimps nei	*Palaemonidae*	0	0	160	110	67	0%	0%	0%	0%	0%
**Penaeus shrimps nei**	***Penaeus spp***	**3450**	**8930**	**21659**	**62132**	**129918**	**38%**	**13%**	**3%**	**5%**	**3%**
**Redtail prawn**	***Penaeus penicillatus***	**0**	**0**	**1769**	**44**	**2**	**0%**	**0%**	**0%**	**0%**	**0%**
**Southern white shrimp**	***Penaeus schmitti***	**0**	**0**	**1206**	**2338**	**0**	**0%**	**0%**	**0%**	**0%**	**0%**
Speckled shrimp	*Metapenaeus monoceros*	0	0	0	0	7533	0%	0%	0%	0%	0%
**Whiteleg shrimp**	***Penaeus vannamei***	***50***	***8359***	**91698**	**154515**	**2690295**	**1%**	**12%**	**13%**	**14%**	**70%**
**Grand total**		8987	71432	680234	1136983	3830247	100%	100%	100%	100%	100%
**Agripenaeina contribution**		7652	61956	648988	1115009	3758987	85%	87%	95%	98%	98%
