# Supplementary material for: The Origin of Large-Bodied Shrimp that Dominate Modern Global Aquaculture
Source: PLoS One. 2016 Jul 14;11(7):e0158840. doi: 10.1371/journal.pone.0158840 (PMC4945062; doi:10.1371/journal.pone.0158840)
Supplement: S5 Table — (PDF) [file pone.0158840.s009.pdf]

S5 Table. Taxa sampled in this study and summary of data collected

Taxonomy*			Molecular (1,480 characters)				Phenomic (339 characters)	
	Genus	Species	H3	NAK	PEPCK	% missing	Specimens§	% missing
**Extant**								
Aristeidae	*Aristaeomorpha*	*foliacea*	GQ487517.1	FJ441125	FJ441170	4.1	139702, 139747	1.7
	Aristaeopsis	*edwardsiana*	JX403895.1	FJ441126	FJ441171	0	255065	0.3
	Aristeus	*virilis*	na	EU427143	EU427212	25.4	288529	0.3
	Hemipenaeus	*carpenteri*	JX403889.1	FJ441140	FJ441185	0	1021925, 1021916, 255812	7.4
	Hepomadus	*tener*	**KX216642**	**KX216650**	**KX216667**	2.8	222645, 236917	6.8
	Parahepomadus	*vaubani*	na	FJ441152	FJ441197	22.2	255846	6.8
	Plesiopenaeus	*armatus*	JX403900.1	FJ441160	FJ441205	0	188970	6.2
	Pseudaristeus	*kathleenae*	na	**KX216651**	**KX216668**	22.2	216716,	9.7
Benthesicymidae	*Bentheogennema*	*intermedia*	JX403892.1	na	**KX216669**	40.3	167604, 167648	19.2
	Benthesicymus	*investigatoris*	na	FJ441131	FJ441176	22.2	1192445,1256413	10.6
	Benthonectes	*filipes*	na	FJ441130	FJ441175	22.3	1256414	8
	Gennadas	*valens*	JX403902.1	**KX216652**	**KX216670**	2	96007	7.4
Penaeidae	*Artemesia*	*longinaris*	**KX216643**	**KX216662**	**KX216681**	0.4	1256144, 1256146, 255567	0
	Atypopenaeus	*formosus*	na	**KX216653**	**KX216671**	33.6	102380, 1256227,102380	6.8
	Atypopenaeus	*stenodactylus*	na	**KX216654**	**KX216672**	23.24	244069	6.2
	Farfantepenaeus	*aztecus*	**KX216647**	FJ441156	FJ441201	1.5	140132	0.6
	Fenneropenaeus	*merguiensis*	na	FJ441159	FJ441204	23.6	1151689	2.4
	Funchalia	*villosa*	JX403893.1	**KX216655**	**KX216673**	2.2	255580	5
	Heteropenaeus	*longimanus*	na	JF899850.1	JF899862.1	25.13	255602	11.8
	Litopenaeus	*setiferus*	JX403906.1	FJ441143	FJ441188	0.1	203506	0.6
	Macropetasma	*africana*	**KX216644**	**KX216656**	**KX216674**	1.6	252426, 216119	6.5
	Marsupenaeus	*japonicus*	na	FJ441145	FJ441190	25.4	216140	0.6
	Megokris	*pescadoreensis*	na	FJ441146	FJ441191	22.2	254367, 156444	7.4
	Melicertus	*latisulcatus*	na	FJ441147	FJ441192	23.5	216150	5.9
	Metapenaeopsis	*palmensis*	na	FJ441153	FJ441198	22.2	255629, 171019	6.2
	Metapenaeus	*ensis*	na	FJ441151	FJ441196	22.2	286927, 156398	5.3
	Parapenaeopsis	*cornuta*	na	FJ441154	FJ441199	22.2	255678	5
	Parapenaeus	*sextuberculatus*	na	FJ441155	FJ441200	22.2	216041	5
	Pelagopenaeus	*balboae*	na	FJ441157	FJ441202	22.3	216499	4.7
	Penaeopsis	*eduardoi*	na	FJ441158	FJ441203	26.5	255511, 255512, 263479	1.8
	Penaeus	*monodon*	na	EU427144	EU427213	25.4	216152	0.9
	Rimapenaeus	*pacificus*	na	FJ441161	FJ441206	22.2	254365, 139217	5.6
	Trachypenaeopsis	*mobilispinis*	na	FJ905029	FJ905030	22.8	251899, 254273	5.6
	Trachysalambria	*curvirostris*	**KX216645**	**KX216657**	**KX216675**	2.6	285366	5.9
	Xiphopenaeus	*kroyeri*	**KX216646**	FJ441168	FJ441213	0.6	254595	5
Sicyoniidae	*Sicyonia*	*brevirostris*	na	**KX216658**	**KX216676**	30.1	225683, 254665	0.6
		laevigata	JX403907.1	**KX216659**	**KX216677**	1.8	254777, 190982	2.1
		lancifer	na	FJ441163	FJ441208	22.2	1011766, 28504	1.5
Solenoceridae	*Cryptopenaeus*	*clevai*	na	FJ441132	FJ441177	22.2	1102156	28.6
	Gordonella	*paravillosa*	na	FJ441136	FJ441181	23.5	266791	6.5
	Hadropenaeus	*lucasii*	na	FJ441137	FJ441182	22.2	253756	6.2
	Haliporoides	*sibogae*	na	FJ441138	FJ441183	25.4	261469	5.3
	Haliporus	*taprobanensis*	na	FJ441139	FJ441184	22.2	253757, 261482	5.3
	Hymenopenaeus	*debilis*	JX403890.1	**KX216664**	**KX216678**	4	253767	9.1
	Hymenopenaeus	*equalis*	na	FJ441142	FJ441187	22.2	258797	6.2
	Mesopenaeus	*brucei*	na	FJ441148	FJ441193	22.2	1102154, 1102155	10
	Pleoticus	*robustus*	JX403901.1	**KX216663**	**KX216682**	0	140617	2
	Solenocera	*melantho*	na	EU427147	EU427216	25.4	254012, 254014	0.9
**Fossils**								
Aegeridae	†*Acanthochirana*	*smithwoodwardii*	na	na	na	100	MSNM i12360, i12241, i12338, i12512, i12394, i12344, i12374	80.2
	†*Aeger*	*tipularius*	na	na	na	100	USNM-PAL-358131, CM-29768	76.7
Penaeidae	†*Antrimpos*	*speciosus*	na	na	na	100	CM 33420, 33375, USNM-PAL-475694, 358134	64.9
	†*Drobna*	*deformis*	na	na	na	100	USNM-PAL-358145, 475720, 475697, 358146, CM-29476, 29466, 29467	67.5
	†*Ifasya*	*madagascariensis*	na	na	na	100	MSNM-i11309, i9311, i9408, i14229, i9383, i9406, i9328, i11243, i9328	84
Benthesicymidae	†*Paleobenthesicymus*	*libanensis*	na	na	na	100	USNM-PAL-358506	70.7
Sergestidae	†*Paleomattea*	*deliciosa*	na	na	na	100	AMNH-44985, 44986, 44987	70.7
**Outgroup**								
Sergestidae	*Deosergestes*	*corniculum*	JX403905.1	**KX216660**	**KX216679**	2.1	258465	1.8
	Sergia	*robusta*	**KX216641**	**KX216661**	**KX216680**	2.4	162071, 165879	5.9
	Sicyonella	*sp.*	**KX216648**	**KX216665**	na	40.1	MNHN-IZ-4990, MNHN-IZ-4046	9.7
	Acetes	*americanus carolinae*	**KX216649**	**KX216666**	na	38.5	258707	5.6
Caridea	*Plesionika*	*grandis*	JF346329	JF346365.1	JF346401	3.1	221415	6.8
	Metalpheus	*rostratipes*	JF346336	JF346372	JF346408	2.9	256799, 256805	11.8
	Systellaspis	*pellucida*	JF346319	JF346355	JF346391	2.9	256513	7.4
Euphausiidae	*Euphasia*	*superba*	na	FJ441133	FJ441178	22.2	1256225	2.1

* Recognized taxonomic groupings and taxonomic assignments are taken from Perez-Farfante and Kensley (1997) and from DeGrave et al. (2009)
§ Catalogue numbers of specimens used for morphology scoring. All extant species were scored from specimens at the USNM (National Museum of Natural History, Smithsonian Institution) unless otherwise specified. Other collection abbreviations are as follows: MNHN: National Museum of Natural History, Paris; CM: Carnegie Museum of Natural History; MSNM: Museo Civico di Storia Naturale di Milano; AMNH: Americal Museum of Natural History; na, not available. Accession numbers for molecular data are from GenBank, with new data generated in this study shown in bold.
