# Supplementary material for: The Origin of Large-Bodied Shrimp that Dominate Modern Global Aquaculture
Source: PLoS One. 2016 Jul 14;11(7):e0158840. doi: 10.1371/journal.pone.0158840 (PMC4945062; doi:10.1371/journal.pone.0158840)
Supplement: S6 Table — (PDF) [file pone.0158840.s010.pdf]

S6 Table. Age information and number of specimens sampled for fossil taxa in this study

Phylogenetic position (this paper, parsimony)	Species sampled by this study	# of specimens examined (museum vouchers)	Number of unambiguous (ambiguous) synapomorphies of smallest clade that includes this fossil	Geology	Age range (mya) *	Median age (mya) *	Ref.
Penaeini	†*Acanthochirana smitwoodwardi*	7 specimens (MSNM i12360, i12241, i12338, i12512, i12394, i12344, i12374)	3 (8)	Hakel, Lebanon, Upper Cretaceous (Cenomanian)	93.9-100.5	97.2	(1)
Parapenaeini	†*Aeger tipularius*	2 specimens (USNM-PAL-358131, CMNH-29768)	2 (4)	Solnhofen area in Germany. Most conservative age is Early Tithonian	145.5-150.8	148.5	(2-5)
Agripenaeina	†*Antrimpos speciosus*	4 specimens (CMNH 33420, 33375, USNM-PAL-475694, 358134)	1 (6)	Solnhofen area in Germany. Most conservative age is Early Tithonian	145.5-150.8	148.5	(2-5)
Trachypenaeini	†*Drobna deformis*	7 specimens (USNM-PAL-358145, 475720, 475697, 358146, CMNH-29476, 29466, 29467)	6 (38)	Solnhofen area in Germany. Most conservative age is Early Tithonian	145.5-150.8	148.5	(2-5)
Parapenaenini	†*Ifasya madagascariensis*	9 specimens (MSNM-i11309, i9311, i9408, i14229, i9383, i9406, i9328, i11243, i9328)	2 (4)	Lower Triassic of Madagascar Griesbachian (Scythian).	247-252	249.5	(6-8)
Benthesicymidae	†*Paleobenthesycimus libanensis*	1 specimen (USNM-PAL-358506)	4 (8)	Sahel Alma, Lebanon. Upper Cretaceous (Santonian)	83.6-86.3	84.95	(1, 9)
Sergestoidea	†*Paleomattea deliciosa*	3 specimens (AMNH-44985, 44986, 44987)	2 (17)	Romualdo Member, Brazil. Lower Cretaceous (Albian)	100.5-113	106.75	(10)

* Age boundaries for each geological range are taken from (11) or are as reported in the references cited for each fossil

1. Garassino A (1994) *The macruran decapod crustaceans of the Upper Cretaceous of Lebanon* (Società italiana di scienze naturali, Museo civico di storia naturale di Milano).
2. Schmid DU, Leinfelder RR, & Schweigert G (2005) Stratigraphy and palaeoenvironments of the Upper Jurassic of Southern Germany–a review. *Zitteliana*:31-41.
3. Schweigert G (2007) Ammonite biostratigraphy as a tool for dating Upper Jurassic lithographic limestones from South Germany–first results and open questions. *Neues Jahrbuch für Geologie und Paläontologie-Abhandlungen* 245(1):117-125.
4. Charbonnier S & Garassino A (2012) The marine arthropods from the Solnhofen Lithographic Limestones (Late Jurassic, Germany) in the collections of the Muséum national d'Histoire naturelle, Paris. *Geodiversitas* 34(4):857-871.
5. Münster Gv & Graf Z (1839) Decapoda Macroura. Abbildung und Beschreibung der fossilen langschwänzigen Krebse in den Kalkschiefern von Bayern. *Beitr. Petrefactenkde* 2:1-88.
6. Van Straelen V (1933) Antrimpos madagascariensis, crustacé décapode du Permotrias de Madagascar. *Bull. Mus. Roy. Hist. Nat. Belgique* 9:1-3.
7. Garassino A & Teruzzi G (1995) Studies on Permo-Trias of Madagascar, III: The Decapod Crustaceans of the Ambilobe Region (NW Madagascar). *Atti della Società italiana di scienze naturali e del museo civico di storia naturale di Milano* 134(1):85-113.
8. Garassino A & Pasini G (2002) Studies on Permo-Trias of Madagascar. 5. Ambilobeia karojoi n. gen., n. sp.(Crustacea, Decapoda) from the Lower Triassic (Olenekian) of Ambilobe region (NW Madagascar). *Atti della Societaitaliana di Scienze naturali e del Museo Civico di Storia naturale in Milano* 143:95-104.
9. Glaessner M (1945) LXX.—Cretaceous Crustacea from Mount Lebanon, Syria. *Journal of Natural History* 12(94):694-707.
10. Maisey J, de Carvalho, M (1995) First Records of Fossil Sergestid Decapods and Fossil Brachyuran Crab Larvae (Arthropoda, Crustacea), with Remarks on Some Supposed Palaemonid Fossils, from the Santana Formation (Aptian-Albian, NE Brazil). *American Museum Novitates* 21:20.
11. Cohen K, Finney S, Gibbard P, & Fan J-X (2013) The ICS international chronostratigraphic chart. *Episodes* 36(3):199-204.
